# Discovery of New Hydrazone-Thiazole Polyphenolic Antioxidants through Computer-Aided Design and In Vitro Experimental Validation

**DOI:** 10.3390/ijms241713277

**Published:** 2023-08-26

**Authors:** Gabriel Marc, Anca Stana, Mihaela Tertiş, Cecilia Cristea, Alexandra Ciorîţă, Ștefan-Mihai Drăgan, Vlad-Alexandru Toma, Raluca Borlan, Monica Focșan, Adrian Pîrnău, Laurian Vlase, Smaranda Oniga, Ovidiu Oniga

**Affiliations:** 1 Department of Pharmaceutical Chemistry, “Iuliu Hațieganu” University of Medicine and Pharmacy, 41 Victor Babeș Street, RO-400012 Cluj-Napoca, Romania; marc.gabriel@umfcluj.ro (G.M.); ooniga@umfcluj.ro (O.O.); 2 Department of Analytical Chemistry, Faculty of Pharmacy, “Iuliu Haţieganu” University of Medicine and Pharmacy, 4 Louis Pasteur Street, RO-400349 Cluj-Napoca, Romania; mihaela.tertis@umfcluj.ro (M.T.); ccristea@umfcluj.ro (C.C.); 3 National Institute for Research and Development of Isotopic and Molecular Technologies, 67-103 Donath Street, RO-400293 Cluj-Napoca, Romania; alexandra.ciorita@itim-cj.ro (A.C.); apirnau@itim-cj.ro (A.P.); 4 Department of Molecular Biology and Biotechnology, Faculty of Biology and Geology, Babeș-Bolyai University, Clinicilor Street No. 4-7, RO-400006 Cluj-Napoca, Romania; stefandragan81@gmail.com (Ș.-M.D.); vlad.toma@ubbcluj.ro (V.-A.T.); 5 Institute of Biological Research, Republicii Street No. 48, Branch of NIRDBS Bucharest, RO-400015 Cluj-Napoca, Romania; 6 Nanobiophotonics and Laser Microspectroscopy Centre, Interdisciplinary Research Institute in Bio-Nano-Sciences, Babeș-Bolyai University, RO-400084 Cluj-Napoca, Romania; raluca.borlan@ubbcluj.ro (R.B.); monica.iosin@ubbcluj.ro (M.F.); 7 Department of Pharmaceutical Technology and Biopharmaceutics, “Iuliu Hațieganu” University of Medicine and Pharmacy, 41 Victor Babeș Street, RO-400012 Cluj-Napoca, Romania; laurian.vlase@umfcluj.ro; 8 Department of Therapeutic Chemistry, “Iuliu Hațieganu” University of Medicine and Pharmacy, 12 Ion Creangă Street, RO-400010 Cluj-Napoca, Romania; smaranda.oniga@umfcluj.ro

**Keywords:** catechol, hydrazone, thiazole, computer-aided design, synthesis, antioxidant, cytotoxicity, electrochemistry

## Abstract

Oxidative stress is linked to a series of diseases; therefore, the development of efficient antioxidants might be beneficial in preventing or ameliorating these conditions. Based on the structure of a previously reported compound with good antioxidant properties and on computational studies, we designed several catechol derivatives with enhanced antioxidant potential. The compounds were synthesized and physicochemically characterized, and their antioxidant activity was assessed through different antiradical, electron transfer and metal ions chelation assays, their electrochemical behavior and cytotoxicity were studied. The results obtained in the in vitro experiments correlated very well with the in silico studies; all final compounds presented very good antioxidant properties, generally superior to those of the reference compounds used. Similarly, the results obtained from studying the compounds’ electrochemical behavior were in good agreement with the results of the antioxidant activity evaluation assays. Regarding the compounds’ cytotoxicity, compound **7b** had a dose-dependent inhibitory effect against all cell lines. In conclusion, through computer-aided design, we developed several catechol thiazolyl-hydrazones with excellent antioxidant properties, of which compound **7b**, with two catechol moieties in its structure, exhibited the best antioxidant activity.

## 1. Introduction

Essentially, oxidative stress results from an alteration of the equilibrium between the production and buildup of reactive oxygen species (ROS) in cells and tissues and the biological system’s ability to immediately neutralize these reactive products and recover after the resulting damage. Oxidative stress can cause cell and tissue breakdown, DNA damage, and is largely considered harmful to the overall health. It is also thought to play an important role in certain illnesses and conditions like diabetes, neurodegenerative diseases, obesity-related disorders, atherosclerosis, cardiovascular diseases, allergic and inflammatory diseases, cancer, etc. Therefore, the use of antioxidants might be beneficial for preventing or reducing the effects of these diseases [1,2,3,4,5].

Polyphenols are natural or synthetic organic compounds of growing interest for the researchers in medicinal chemistry due to their antioxidant and anti-inflammatory properties, which could be exploited for the prevention and treatment of many diseases linked to oxidative stress [6,7,8]. Polyphenols’ biological properties depend strongly on their chemical structure, the number of hydroxyl groups influencing certain mechanisms of antioxidant activity like free radical scavenging, metal ions chelation ability, inhibition of ROS production by suppressing the enzymes involved in their production or upregulating the antioxidant defenses [6,9].

Starting from a previously synthesized catechol derivative, 2-(3,4-dihydroxybenzylidene)-1-(4-methylthiazol-2-yl)hydrazin-1-ium chloride (CHT) [10], which presented very good antioxidant properties, we designed, based on in silico and thermodynamic calculations, several polyphenolic molecules with similar structures to that of CHT. For the compounds’ design, molecular hybridization was used, based on combining the most suitable pharmacophore moieties, in order to obtain compounds with enhanced antioxidant activity. As hydrazone derivatives were reported to possess antioxidant activities [11,12], a hydrazone moiety was included in the structure of the final compounds as it can release hydrogen atoms, contributing to the antioxidant potential of the compounds. Numerous research papers also report the use of 2,3-dihydroxybenzaldehyde thiosemicarbazones as ligands for metal ions [13,14,15]. Taking into account these findings and the fact that the polyphenolic derivatives we reported in previous research either lacked or possessed modest metal ions chelating properties [16,17], we exploited the possibility of introducing a hydroxyl group in position 2 of the benzylidene fragment, with the scope of improving the metal ions’ chelation ability of compounds. In addition, we envisioned to determine whether the biological potential of the compounds is altered by the steric hindrance in the 2,3-dihydroxybenzylidene derivatives, compared to the 3,4-dihydroxybenzylidene derivatives.

Several in vitro experiments were used to assess the antioxidant capability of the novel compounds. These experiments include assessments such as radical scavenging, electron transfer, and metal ion chelation assays, alongside an investigation into their electrochemical properties. Then, the newly synthesized compounds were subjected to cytotoxicity assays targeting both normal and cancer cell lines, wherein assessments of cell viability and subsequent membrane integrity were conducted, and the cells’ response to oxidative stress was determined.

## 2. Results and Discussion

### 2.1. Validation of the Design Hypothesis Using In Silico and Thermodynamic Calculations

Based on previous reports in the literature and our group’s experience in this field, the easiness with which hydrogen atom transfer (HAT) can occur was evaluated, expressing the results as bond dissociation enthalpy (BDE). The ease with which the molecules’ phenolic groups release hydrogen atoms was evaluated, as well as the hydrazone group, which according to our previous research and that of other researchers, can discharge hydrogen atoms into the environment, acting as an antioxidant. Other groups in the proposed molecules, which contain a hydrogen atom that could be released (such as the azomethine group or the 5-position of the thiazole nucleus), were not considered in the present study, because in our previous research they presented very high BDEs, indicating that it is unlikely to extract a hydrogen atom from those sites [17,18]. 

The outcomes of the screening process for the library of proposed analytes are presented in Table 1. The BDE for the O-H and N-H homolytic breaking was computed in vacuum to identify the desirable substituents on the thiazole ring.

Analysis of the BDE of O-H and N-H indicated that the electron withdrawing groups, such as esters or ketones, are negatively influencing the release of hydrogen atoms from both series of proposed compounds. Electron donating groups are accepted only if they are small in volume and not simultaneously present in positions 4 and 5 of the thiazole ring. For the same type of substituent, the two series of compounds did not have the same behavior in terms of easiness of hydrogen atom release. Mainly, this was driven by the apparition of the intramolecular hydrogen bonding between the phenol from the ortho position and hydrazone bridge. Furthermore, it was observed that the insertion of a phenyl ring in position 4 of the thiazole ring would be beneficial for the antiradical activity of the compounds. If in addition to the phenyl ring from position 4 of the thiazole ring, another group could be grafted in position 5 of the thiazole ring; this would negatively affect the activity in the series **b** of compounds. Since the substitution with a phenyl ring in position 4 of the thiazole is favorable to the compounds’ antioxidant activity, we proposed to graft a catechol in that position, which is actually a phenyl ring that comes with the advantage of having an additional catechol group in the molecule, bringing increased antioxidant activity for the resulting compounds than if there was just a phenyl in that position. Therefore, we have abandoned studying molecules just with a phenyl in position 4 of the thiazole, since we had a much better alternative available.

Overall, the studies reported in the present paper were continued on two types of substitutions, both in position 4 of the thiazole nucleus: one with a methyl group and the other with a catechol group.

Since the solvent can influence the behavior of the reactants in a chemical reaction, the BDE evaluation study was carried out using several environments: vacuum, a non-polar solvent (toluene), a medium polarity solvent (ethanol) and a high-polarity solvent (water). 

**Figure 1:**
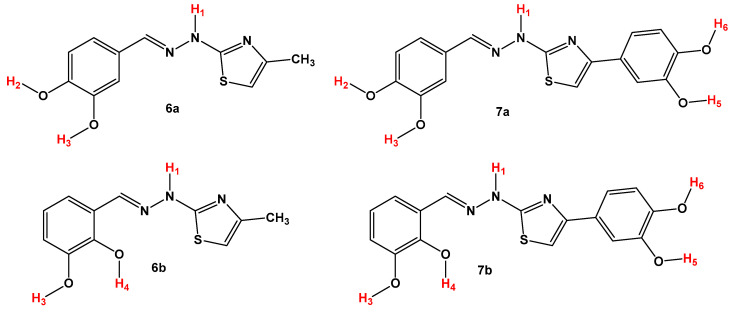
General structure of the proposed compounds **6a–b** (left) and **7a–b** (right) and their potential locations from which hydrogen atoms could be released to counteract external radicals.

Figure 1 illustrates the chemical structures of the suggested compounds, highlighting the positions capable of hydrogen atom donation. Results of the BDE calculations for each molecular site from Figure 1 are presented in Table 2, Table 3, Table 4 and Table 5.

The BDE for the N-H bond of the hydrazone group (H_1_) varied in an interesting way in the two series of compounds, the compounds from series **b** exhibiting lower BDEs than those of series **a**, regardless of the solvent used. Within the **a** series of compounds (3,4-dihydroxybenzylidene derivatives), the N-H_1_ BDE varied slightly between 67.85 kcal/mol and 69.80 kcal/mol, mainly independently of the substituent in position 4 of the thiazole nucleus, with lower values being identified in toluene compared to the other solvents. In series **b** of the compounds, BDE varied more widely, depending on the substitution on the thiazole nucleus and the solvent, between 63.55 kcal/mol and 66.53 kcal/mol. Analysis of the substituents’ influence on BDE indicated that the best substitution for lowering the BDE of the N-H from hydrazone was membership to the 2,3-dihydroxybenzylidene series and the presence of a catechol moiety in position 4 of the thiazole ring.

Related to the benzylidene fragment from the compounds structure that could release hydrogen atoms (sites H_2_, H_3_ and H_4_), the H_4_ site stands out by far, with the lowest BDE values between 62.10 kcal/mol and 65.32 kcal/mol. This can be explained by the possibility of forming bonds with the hydrazone fragment in the vicinity of the phenolic group, with the obtaining of pseudocyclic structures that favor the stabilization of the resulting radical. If the radicalization takes place at the level of this site, the phenolic group in meta (the O-H_3_ site) could intervene through a hydrogen bond, contributing to the stabilization of the obtained radical. These two corroborated contributions explain the much lower breaking energy of O-H_4_ compared to the other sites on the benzylidene moiety. Regarding the most active site within series **a** of the compounds, things became strongly dependent not only to the substitution, but also to the solvent. In vacuum, the most active site in this series was H_3_ in non-polar solvents or in solvents with medium polarity. According to the increase in solvent polarity, the BDE of O-H_2_ bond decreased, becoming even smaller than the O-H_3_ BDE. 

Regarding sites H_5_ and H_6_, the most favorable to release hydrogen atoms was site H_6_, with a BDE ranging from 65.45 kcal/mol to 69.96 kcal/mol, significantly lower to those from the site H_5_, ranging from 76.35 kcal/mol to 78.91 kcal/mol. The solvent effect was mainly identified for site H_6_, while the solvent effect on site H_5_ was much lower. BDE of the O-H_6_ bond increased slightly with the increase in solvent polarity but, considerably, this is not able to affect the antiradical activity of this group.

Comparing the computed BDEs for the proposed structures with our previously reported results suggested a sustainable working hypothesis. All our previous BDE calculations were made in vacuum, therefore comparison between our previous results and current results will be limited to the BDEs computed in vacuum (Table 2). Compared with our previous reports (Table 6), it is shown that the lowest BDEs identified in the respective papers are similar to those presented in Table 2, suggesting that the proposed structures would act as strong antioxidants.

### 2.2. Chemical Synthesis

The intermediate thiosemicarbazones **3a–b** were obtained by the condensation of 3,4-dihydroxybenzaldehyde (**1a**) or of 2,3-dihydroxybenzaldehyde (**1b**) with thiosemicarbazide (**2**) in a slightly acidic environment, following previously reported protocols in the scientific literature for the synthesis of other thiosemicarbazones [12]. 

The Hantzsch heterocyclisation of thiosemicarbazones **3a–b**, accompanied by the relevant α-chloroketones (**4** or **5**), provided the desired final products. When chloroacetone (**4**) was used as the heterocyclisation reagent of the thiosemicarbazones **3a–b**, the final compounds **6a–b** were obtained, while using 4-chloroacetyl-catechol (**5**) resulted in the final compounds **7a–b**. All final compounds were isolated from the reaction environment as hydrochlorides, which precipitated from the reaction environment and were filtered by suction. The resulting crude solid was later crystallized from acetone to provide the pure final products **6a–b** and **7a–b**. Compounds **3a** and **6a** were previously reported [9].

For all intermediate and final compounds, the corresponding molecular peak was identified in the mass spectrum. A fragmentation pattern due to the breaking of the N-N bond from the hydrazone moiety was identified for all intermediate and final compounds. In all compounds a peak at *m/z* ≈ 134 can be identified, corresponding to the iminomethyl-catechol fragment, while compounds **7a–b** depicted a supplementary peak at *m/z* = 206.9 (for **7a**) and at *m/z* = 206.8 (for **7b**), which was provided by the 4-(2-aminothiazol-4-yl)-catechol fragment. 

The infrared spectra recorded for the compounds indicated their successful obtention with the expected signals for the phenol groups, N-H and C=N bonds in the corresponding regions.

In the proton nuclear magnetic resonance (^1^H-NMR) spectra of the compounds, all expected signals provided by the protons were spotted, with the corresponding multiplicity and the associated coupling constants. The most deshielded proton was identified to be azomethine, appearing as a singlet between 8.008 ppm and 8.601 ppm. A characteristic for the successful synthesis of the final compounds after the Hantzsch cyclisation was the appearance of the proton from the C5 of the thiazole ring as a singlet between 6.617 ppm and 6.967 ppm. For compound **6b** the three protons from the methyl, which grafted on C4 of thiazole ring, appeared as a singlet at 2.226 ppm.

In the carbon nuclear magnetic resonance (^13^C-NMR) spectra of the final compounds, the C2 atom from the thiazole ring appeared between 167.310 ppm and 168.297 ppm, as the most deshielded. The carbon atoms carrying the phenol groups and the azomethine carbons were identified between 159.059 ppm and 149.028 ppm. In the aromatic region of the ^13^C-NMR spectra, the other aromatic carbon atoms, from the two benzene rings were identified between 112.638 ppm and 125.524 ppm. The peaks given by the C5 of the thiazole ring were identified between 100.537 ppm and 103.098 ppm, thus confirming the successful synthesis of the desired compounds, particularly for compound **6b**, the carbon atom from the methyl group was identified at 14.404 ppm. 

### 2.3. In Vitro Antiradical, Electron Transfer and Metal Ions Chelation Assays

#### 2.3.1. Antiradical Assays

The capacity of compounds **6a–b** and **7a–b** to scavenge radicals 1,1-diphenyl-2-picrylhydrazyl (DPPH^•^), 2,2′-azino-bis(3-ethylbenzothiazoline-6-sulfonic acid) (ABTS^•+^) and ^1^O_2_ is presented in Table 7 and Table 8, along with their half-maximal inhibitory concentrations (IC_50_). 

In both DPPH^•^ and ABTS^•+^ scavenging assays, the most active compound was found to be **7b** (IC_50 DPPH_^•^ = 4.85 µM and IC_50 ABTS_^•+^ = 2.55 µM, respectively). Comparing the results obtained for the two series of compounds, it is obvious that in the DPPH^•^ and ABTS^•+^ scavenging assay compounds from the **b** series (2,3-dihydroxybenzylidene) were more active than those from the **a** series (3,4-dihydroxybenzylidene). Comparing the activity of the studied compounds according to the substitution of the thiazole ring in position 4, it is shown that both substitutions (methyl and catechol) are favorable in terms of antiradical activity. The most active compounds were those substituted with a catechol moiety in position 4 of the thiazole (**7a–b**).

**Figure 2:**
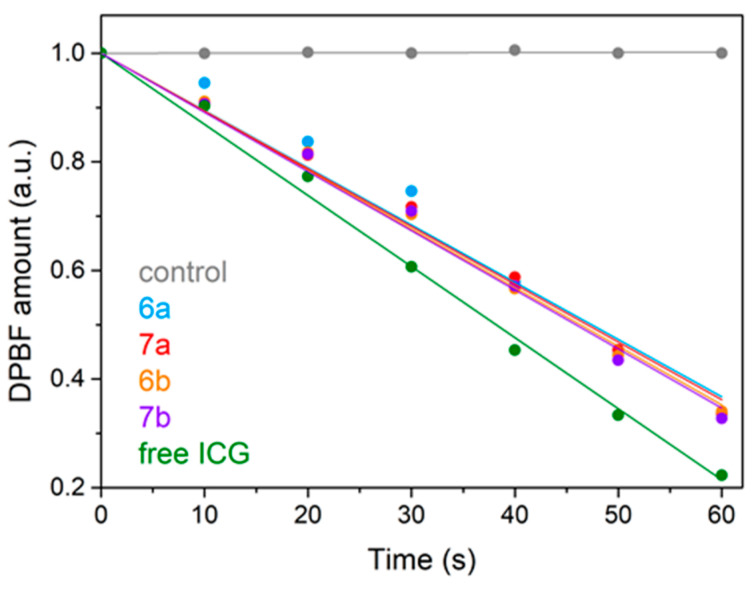
The absorption of DPBF at 419 nm in the presence of ICG and **6a** (black), **6b** (orange), **7a** (red), and **7b** (violet) measured after 0, 10, 20, 30, 40, 50 and 60 s of irradiation.

**Figure 3:**
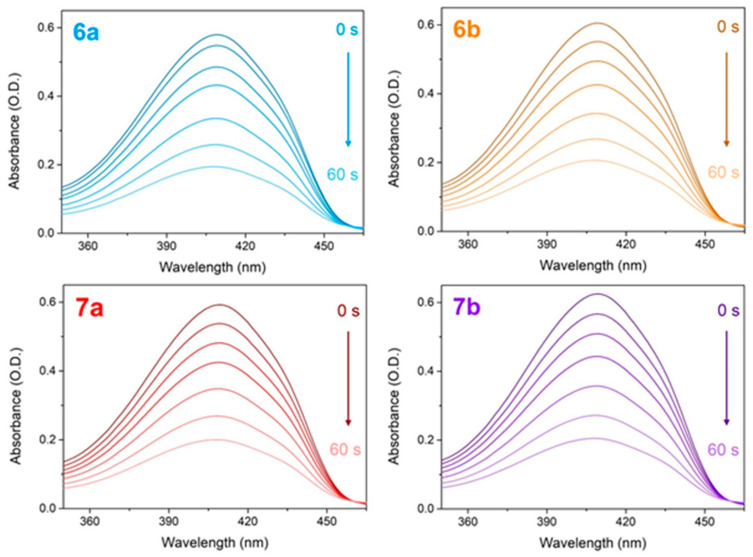
Absorption spectra of DPBF in the presence of ICG and **6a** (blue), **6b** (orange), **7a** (red), and **7b** (violet) measured after 0, 10, 20, 30, 40, 50 and 60 s of irradiation.

As previously reported by our group, compound **6a** has promising ^1^O_2_ scavenging capacity correlated with reference compounds [10]. Thus, in this current investigation, our emphasis was directed towards comparing the scavenging activity of the compounds using the quantum yield of ^1^O_2_ production of free indocyanine green (ICG) as a reference. Figure 2 and Figure 3 present the degradation of 1,3-diphenylisobenzofuran (DPBF) in the presence of the ^1^O_2_ generator, ICG, and the tested compounds, highlighted by the decrease in the optical density of the sensor monitored after every 10 s of irradiation. Based on these measurements, the quantum yields of ^1^O_2_ production were calculated for all four compounds using the ^1^O_2_ quantum yield of free ICG as a reference [10,20]. Since a lower quantum yield of ^1^O_2_ production of a molecule can be interpreted as having a better ^1^O_2_ scavenging capacity, all studied molecules displayed good antioxidant activity and, between them, **7a** presented the highest activity as an ^1^O_2_ scavenger.

#### 2.3.2. Electron Transfer Assays

The outcomes of the electron transfer assays are displayed within Table 9. The activity of the compounds evaluated in the redox assays followed an identical trend, regardless of the assay applied. Except for the compound **6a** in the Total Antioxidant Capacity (TAC) assay, its equimolar activity is lower than that of trolox. In all other assays, each compound exhibited higher activity than those of the reference compounds at the same molar concentration.

The analysis of the results obtained in the electron transfer assays indicates that the compounds from the **b** series were more active than those from the **a** series, and series **7** were more active than those of series **6**. Overall, the activity of the compounds in the electron transfer assays has the following trend: **6a** < **7a** < **6b** < **7b**.

#### 2.3.3. Assays for Metal Ions Chelation

The results regarding the chelation capacity of ferrous and cupric ions are presented in Table 10 and Table 11.

A low capacity for chelating ferrous ions was identified for compounds **6b**, **7a,** and **7b** at high concentrations. Their activity is much lower than that of ethylenediaminetetraacetic acid disodium salt (EDTA-Na_2_).

The cupric ions chelation activity is quite similar to EDTA-Na_2_, but no clear structure-activity relation can be identified; all studied compounds exhibited similar cupric ions chelation activity.

Of the four synthesized compounds, **7b** presented the best chelating properties for both ferrous and cupric ions, although it was inferior to those of the reference used in the assays. 

### 2.4. Electrochemical Behavior of Compounds

The antiradical and/or antioxidant capacity/activity of a compound is correlated with the values of oxidation potentials recorded in its presence using electrochemical methods, such as cyclic voltammetry and electrochemically inert electrodes. Hence, lower values indicate a compound’s suitability for electron donation, which corresponds to substantial antioxidant/antiradical effectiveness. When oxidation potential values are closely matched, compounds featuring higher oxidation peak current intensity will exhibit greater antioxidant capacity.

**Figure 4:**
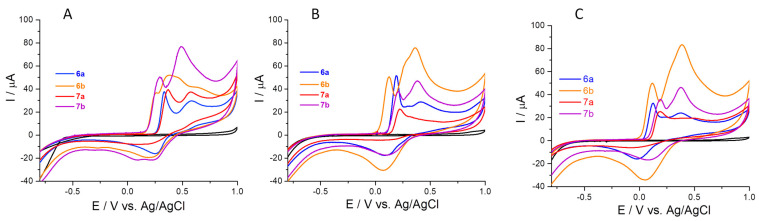
Cyclic voltammograms for 0.5 mM solutions of: **6a** (blue); **7a** (red); **6b** (orange); and **7b** (violet) on graphite-based screen-printed electrodes. Different electrolyte solutions were used such as 0.1 M HClO_4_ (**A**); 0.1 M AB with pH = 4 (**B**); and BRB with pH = 4 (**C**), and the voltammogram of the corresponding electrolyte is depicted with black color.

The cyclic voltammograms of the compounds were plotted in different electrolytic media on screen-printed electrodes, with carbon as a working electrode, in order to compare the position of the oxidation/reduction signals and evaluate their magnitude. In Figure 4, the cyclic voltammograms (CVs) for each compound are presented, juxtaposed individually with the voltammogram acquired in the electrolyte, namely in the presence of 0.1 M HClO_4_ solution with pH = 1.2 (Figure 4A), 0.1 M acetate buffer (AB) with pH = 4 (Figure 4B) and 40 mM of Britton–Robinson buffer (BRB) with pH = 4 (Figure 4C). Therefore, both the oxidation and reduction peaks corresponding to the electrochemical changes experienced by each compound can be observed within the specified potential range at a strongly and moderately acidic pH.

**Figure 5:**
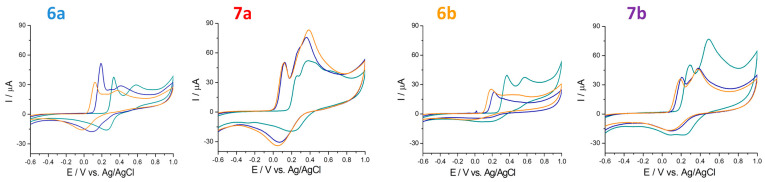
Cyclic voltammograms for 500 μM solutions of: **6a**; **7a**; **6b** and **7b** prepared in 0.1 M HClO_4_ (green); 0.1 M AB with pH = 4 (blue); and BRB with pH = 4 (orange).

From Figure 4, illustrating the voltammograms of all four compounds, a similarity between **7a** and **7b** can be observed, regardless of the type of electrolyte used. These two compounds presented oxidation potentials with lower values, and higher values of intensity. The differences between the values of the oxidation and reduction potentials for the four analytes are presented in Table 12 and Figure 5, where the voltammograms of the same compound, registered in different electrolytic media, are compared. Table 12 presents, for each compound, the values from the voltammograms obtained at two different concentrations for each analyte: 250 and 500 μM.

#### 2.4.1. Antioxidant Capacity Determined through Ferric Ions (Fe^3+^)

The reducing potential of the end products can be ascertained using a common method based on the reducing capacity of Fe^3+^. Thus, the ability to reduce of an analyte tested on Fe(III) ions indicates the capacity to donate electrons of that compound and is linked to antioxidant capacity.

This assay relies on the conversion of Fe^3+^ to Fe^2+^ brought about by the action of antioxidants, and involves evaluating the analyte’s influence, evaluated through the direct reduction of Fe[(CN)_6_]^3−^ to Fe[(CN)_6_]^2−^. Drawing from the electrochemical characteristics of the investigated compounds, the interaction with Fe^3+^ was tracked using cyclic voltammetry. This involved monitoring potential shifts in the redox peaks that signify the Fe^3+^/Fe^2+^ transition, subsequent to the swift interaction with the tested compounds (the tests were performed only 1 min after the contact of each new compound with the solution containing Fe(III) ions).

**Figure 6:**
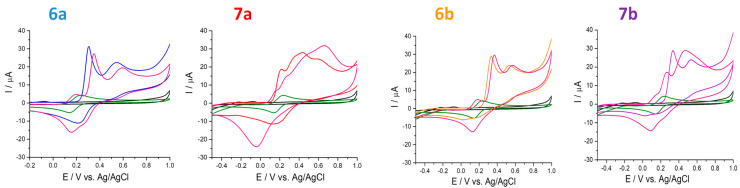
Cyclic voltammograms obtained in the presence of: 500 μM potassium ferricyanide (green); 250 μM solution containing the novel synthesized substances (different color based on the compound) and of solutions containing 500 μM ferricyanide and 250 μM of a single compound at a (pink) in 0.1 M HClO_4_ registered by using the graphite screen-printed electrodes (SPEs): **6a **(blue), **7a** (red), **6b** (orange) and **7b** (violet). The voltammogram of 0.1 M HClO_4_ electrolyte is depicted using black.

Figure 6 shows the voltammogram of 500 μM potassium ferricyanide performed in the presence of 0.1 M HClO_4_ (green) of each analyte at a concentration of 250 μM prepared in 0.1 M HClO_4_ (different color based on the compound) contrasted with that of the mixtures between potassium ferricyanide (500 μM) and a specific analyte (250 μM) (pink). The voltammogram of potassium ferricyanide reveals an evident one-electron transfer process that occurs for Fe^3+^/Fe^2+^ at a potential of about 0.235 V/0.140 V under acidic condition(green) (Figure 6; Table 13).

Each analyte considered in this study exhibits distinct characteristic signals (different color based on the compound), and when the mixtures were examined (pink), a reduction in the characteristic redox peaks associated with the Fe^3+^/Fe^2+^ process signifies interaction with a compound. This interaction suggests that the compound displays a reduction action towards the ferricyanide ion, indicating antioxidant capacity. Simultaneously, the oxidation peaks of analytes can be impacted by the presence of ferricyanide in the solution. They undergo partial oxidation during the interaction with Fe^3+^, causing the electrochemical oxidation to be slower, as evidenced by the shift of anodic signals towards higher potentials.

An alternative pattern can be noted once more for each compound, however, once more, **7a** exhibited a pattern comparable to **7b**, while **6a** can be compared to **6b**. These compounds were comparatively depicted individually and in the presence of the redox probe as cyclic voltammograms in Figure 6.

The alterations in potential and intensity of the oxidation currents among different compounds are depicted in Table 13. It is observed that all compounds caused a shift of the oxidation signal of ferricyanide to smaller values (in the cathodic way) simultaneously with a reduction in the magnitude of the oxidation current for this compound. This decrease was more accentuated in the case of **7b** (by 91.2%), followed by **7a** (by 75.86%), then **6a** (by 44.47%) and **6b** (by 36.34%). This behavior suggests that **7b** has the most pronounced scavenging effect on ferricyanide, followed by **7a**, **6a**, and **6b**. This type of influence on the redox probe is normal and is reported in the literature [17,21]. This phenomenon usually takes place concurrently with the elevation of the compounds’ oxidation peaks.

Compound **7b** had the most significant scavenging effect on ferric ions. It was noticed that the fluctuations in signals the oxidation peaks of the studied analytes did not validate the observed pattern in the redox probe signal. The normal tendency would be for the oxidation peaks of the analytes that cause the reduction in the ferricyanide signal to increase. In reality, they have decreased, more or less, from one compound to another. A small increase from 10.13 μA to 13.58 μA in the oxidation signal of **7a** was, however, observed. An explanation for the unexpected trend could arise from the fact that nearly all analytes exhibit a signal within the potential range of the ferric ions signal. Consequently, there is a potential for interference between these signals. The same behavior was previously reported in a study conducted by our team on other organic compounds [17].

#### 2.4.2. Hydrogen Peroxide Scavenging

Hydrogen peroxide (H_2_O_2_) is not inherently a free radical, but it readily transforms into free radicals such as OH^•^ within biological systems. In this context, it becomes a highly destructive agent in free radical-related issues, capable of harming virtually every molecule within living cells.

The anodic redox transformation of H_2_O_2_ was previously reported to assess the antioxidant nature of different analytes in beverages. This approach was also employed to track variations in the oxidation signal of H_2_O_2_ caused by the introduction of varying quantities of piperine, along with other organic compounds [17,22,23].

Gold-based, screen-printed electrodes (SPEs), provided by Metrohm Dropsens (Oviedo, Asturias, Spain), served as a platform for observing alterations in the oxidation signal of H_2_O_2_, consecutively following the addition of various quantities of the novel synthesized substances.

**Figure 7:**
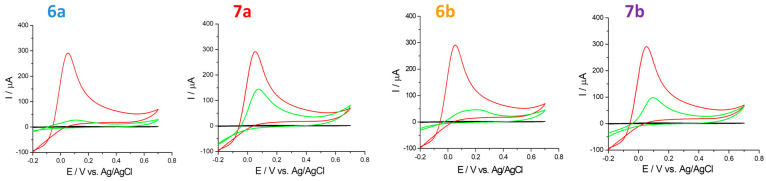
Cyclic voltammograms registered on gold SPEs for: 0.1 M KCl as electrolyte (black); for 500 μM of H_2_O_2_ in 0.1 M KCl (red). Cyclic voltammogram drawn in green corresponds to the tests performed 1 min after contacting 250 μM of the new compounds with 500 μM H_2_O_2_ prepared in 0.1 M KCl, registered using gold-based SPEs: **6a**; **7a**; **6b** and **7b**.

The oxidation peak of H_2_O_2_, acquired within the potential range of −0.5 to 1.2 V on the chosen gold electrode, is presented in Figure 7, compared to that obtained in the absence of the analyte using 0.1 M KCl (black in all four graphs). 

Usually, the addition of an antioxidant in a solution containing H_2_O_2_ results in the reduction of the anodic peak distinctive to H_2_O_2_. It can be inferred that antioxidants operate by interacting with dissociated H_2_O_2_, leading to a decline in its concentration. This, in turn, causes a decrease in the magnitude of the anodic signal, which correlates with antioxidant capacity. However, this suggests that the antioxidant needs to interact with the present H_2_O_2_ in the solution.

Various theories were proposed in the literature to support the significance of specific structural elements as requisites for radical scavenging and antioxidant effects. Most of these theories revolve around aspects like configuration, substitution, and the number of hydroxyl groups.

Table 14 presents the changes in the potential and magnitude of the oxidation current 500 μM H_2_O_2_ after just one minute of contact with one compound. It is observed that all compounds caused a shift of the oxidation potential of H_2_O_2_ to higher values (in the anodic way) simultaneously, with a decrease in the value of the intensity of the oxidation current for H_2_O_2_. This decrease was more accentuated in the case of **6b** (by 87.14%), followed by **6a** (by 86.68%), then **7b** (by 69.00%) and **7a** (by 48.09%). This behavior suggests that **6b** has the most pronounced scavenging effect on H_2_O_2_, followed by **6a**, **7b**, and **7a**, which contradicts the results presented above, which were obtained in the presence of potassium ferricyanide.

Attempts were made to increase the concentration of the synthesized compounds to 500 μM, in order to verify the observations made using solutions containing 250 μM of each analyte in the presence of 500 μM H_2_O_2_. In this case, however, the oxidation signal of hydrogen peroxide disappeared completely, which did not allow us to draw a different conclusion from the initial one.

#### 2.4.3. The 1,1-Diphenyl-2-picrylhydrazyl (DPPH^•^) Free Radical Scavenging

The scavenging capabilities against the radicals exhibited by the synthesized catechol derivatives and other antioxidants (ascorbic acid), but also of a compound that is known not to have antioxidant capacity (tyrosine), were assessed in the presence of a stock solution of DPPH^•^ prepared in absolute ethanol. The solutions containing the novel compounds were combined with the DPPH^•^ stock solution and HClO_4_ electrolyte and kept in dark for 30 min and 120 min, respectively. The residual redox reactivity of DPPH^•^ was evaluated by cyclically altering the potential within the range of −0.7 V to 1.2 V, employing a scan rate of 100 mV s^−1^.

**Figure 8:**
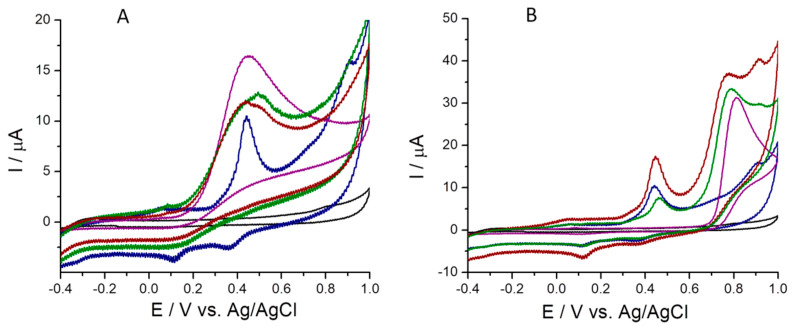
Cyclic voltammograms performed on graphite-based SPEs using a solution containing 500 μM DPPH^•^ prepared with 0.1 M HClO_4_ (blue). The voltammogram of 0.1 M HClO_4_ electrolyte is depicted by black color. (**A**) Ascorbic acid effect on DPPH^•^. Cyclic voltammograms registered in the presence of: 500 μM ascorbic acid (purple); 500 μM ascorbic acid + 500 μM DPPH^•^ after a 30 min reaction conducted in the absence of light (dark red); 500 μM ascorbic acid + 500 μM DPPH^•^ after 120 min of reaction performed in the dark (green). (**B**) Tyrosine effect on DPPH^•^. Cyclic voltammograms registered with: 500 μM tyrosine (purple); 500 μM tyrosine + 500 μM DPPH^•^ after a 30 min reaction conducted in the absence of light (dark red); 500 μM tyrosine + 500 μM DPPH^•^ after a 120 min reaction conducted in the absence of light (green). All the solutions were prepared using 0.1 M HClO_4_.

The nitrogen-centered DPPH^•^ radical-based configuration is among the most commonly utilized techniques to assess a compound’s capacity for scavenging or neutralizing free radicals. The electrochemical features of DPPH^•^ were investigated using graphite-based SPEs in HClO_4_ (Figure 8A,B); navy). The findings indicated that DPPH^•^ experiences reversible reduction and oxidation, evident from the redox peak couple positioned at 0.442 V/0.360 V.

Considering the redox behavior of DPPH^•^, its reduction, due to the presence of an antioxidant, can also be tracked using cyclic voltammetry, comparing the oxidation/reduction signals before and after the interaction with the target compounds. Figure 8 reports the electrochemical behavior of DPPH^•^ during the electrochemical oxidation (blue) and the selected control compounds having known antioxidant activity: ascorbic acid (Figure 8A), which has good antioxidant capacity; and tyrosine (Figure 8B), a compound without antioxidant activity. Details on the correspondence between color and voltammograms are included in the explanatory title of Figure 8.

It was observed that the signal of DPPH^•^ and ascorbic acid appeared in the same potential range, so it is very difficult to follow the signal individually when they are together in the solution (Figure 8A). However, the decrease in ascorbic acid signal after 30 min of reaction with DPPH^•^ proved the existence of this interaction. Increasing the reaction time to 120 min led to no changes in the appearance of the voltammogram of ascorbic acid, proving the complete consumption of the radical in the solution in the first 30 min of contact with the antioxidant control agent.

In the case of tyrosine (Figure 8B), another control compound, it was observed that the position and intensity of the radical oxidation/reduction signal were only slightly influenced by the presence for 30 and 120 min of this compound, respectively. It can be considered that there was no interaction between DPPH^•^ and tyrosine, thus having a behavior example of each kind, with and without antioxidant action, is very useful to evaluate the properties of new compounds.

**Figure 9:**
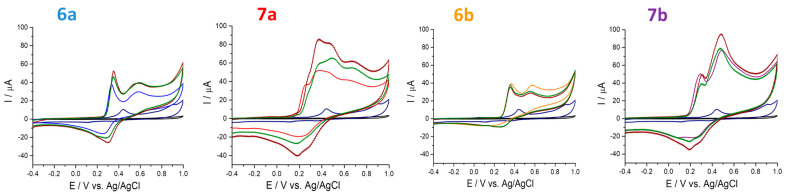
Cyclic voltammograms obtained using graphite-based SPEs with: 500 μM DPPH^•^ in 0.1 M HClO_4_ (blue); 500 μM solution of the new analytes (different color based on the compound) and of samples containing 500 μM DPPH^•^ and 500 μM of one compound at a time: after 30 min (dark red) or 120 min (green) reaction in the dark. **6a**; **7a**; **6b** and **7b**. All the solutions were prepared using 0.1 M HClO_4_.

After studying the voltammograms obtained for the solutions of the final compounds alone in the solution, at a given concentration, or together with the DPPH^•^ radical at the same concentration, it was observed that after 30 and 120 min of reaction, respectively, there were significant changes only in case of **7a** and **7b** (Figure 9), which can be correlated with their interaction with the radical. Thus, we can conclude that **7a** and **7b** have better antioxidant capacity than **6a** and **6b** (Figure 9).

#### 2.4.4. 2,2,6,6-Tetramethylpiperidinyl-N-oxyl (TEMPO) Scavenging

**Figure 10:**
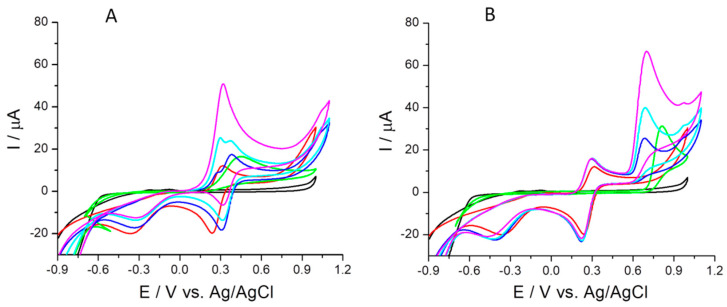
Cyclic voltammograms obtained using graphite-based SPEs in: 2 mM TEMPO prepared in 0.1 M HClO_4_ (red) compared with: (**A**) Ascorbic acid: ascorbic acid 500 μM in HClO_4_ (green); TEMPO 2 mM + ascorbic acid 250 μM after a 60 min reaction conducted in the absence of light (dark blue); TEMPO 2 mM + ascorbic acid 500 μM after a 60 min reaction conducted in the absence of light (light blue); TEMPO 2 mM + ascorbic acid 1 mM after a 60 min reaction conducted in the absence of light (pink); and (**B**) Tyrosine: tyrosine 500 μM in HClO_4_ (green); TEMPO 2 mM + tyrosine 250 μM after 60 min reaction in the dark (dark blue); TEMPO 2 mM + tyrosine 500 μM after a 60 min reaction conducted in the absence of light (light blue); TEMPO 2 mM + tyrosine 1 mM after a 60 min reaction conducted in the absence of light (pink). The voltammogram of 0.1 M HClO_4_ electrolyte is depicted using black color.

2,2,6,6-tetramethylpiperidine-N-oxyl is a stable organic nitroxide radical documented for its ability to scavenge carbon-centered radicals. The cyclic voltammograms of a 2 mM TEMPO solution in HClO_4_ (Figure 10; red line) highlighted its reversible one-electron transfer process. This was evident from the oxidation signal at 0.301 V, indicative of the oxidation of TEMPO to oxoammonium species, coupled with a cathodic peak at 0.232 V that corresponds to the reduction of electrochemically produced oxoammonium species.

Cyclic voltammetry was subsequently employed to assess the antioxidant efficacy of the synthesized catechol derivatives by their capacity to neutralize the free radical species. Figure 10A presents the overlapped voltammograms registered in the presence of 2 mM TEMPO and 2 mM TEMPO after a reaction conducted in the dark for 60 min in the presence of diverse concentrations of ascorbic acid: 250 μM (dark blue); 500 μM (light blue); and 1 mM (pink). It is evident that the oxidation signal of TEMPO decreased dramatically from 13.81 μM to 1.44 μM, after 60 min of reaction with 250 μM ascorbic acid, used as the control antioxidant. When increasing the concentration of ascorbic acid to 500 μM and 1 mM, respectively, the intensity of the signals recorded in the oxidation potential range of TEMPO increased. In fact, the oxidation signal, whose intensity increased, was that of ascorbic acid, which remained unreacted after the full consumption of TEMPO in the solution, given that it was consumed almost entirely (about 90%) in the reaction with the lowest concentration of ascorbic acid. It was also noticed that the intensity of the electrochemical reduction of oxoammonium species of TEMPO, which occurred at 0.232 V, showed no change, even at high concentrations of ascorbic acid, but only slightly shifted anodically. These observations confirm that the entire amount of TEMPO was oxidized in the reaction with ascorbic acid, while during the potential scan in the cathodic direction, the oxidation product was reduced by reforming the initial concentration of TEMPO. Figure 10B shows the overlaid voltammograms obtained in the presence of 2 mM TEMPO and 2 mM TEMPO contacted for 60 min in the dark with solutions of tyrosine of: 250 μM (dark blue); 500 μM (light blue); and 1 mM (pink). This compound was used because it was shown to lack antioxidant behavior, being used as a control to prove that the TEMPO concentration does not change significantly if there are no compounds with antioxidant capacity in the solution. It can be observed that the oxidation/reduction signals of TEMPO did not significantly influence its values, even in contact with high concentrations of tyrosine, which proves the above-mentioned assumption. The only signal that increased when increasing the tyrosine concentration was that corresponding to its electrochemical oxidation. 

**Figure 11:**
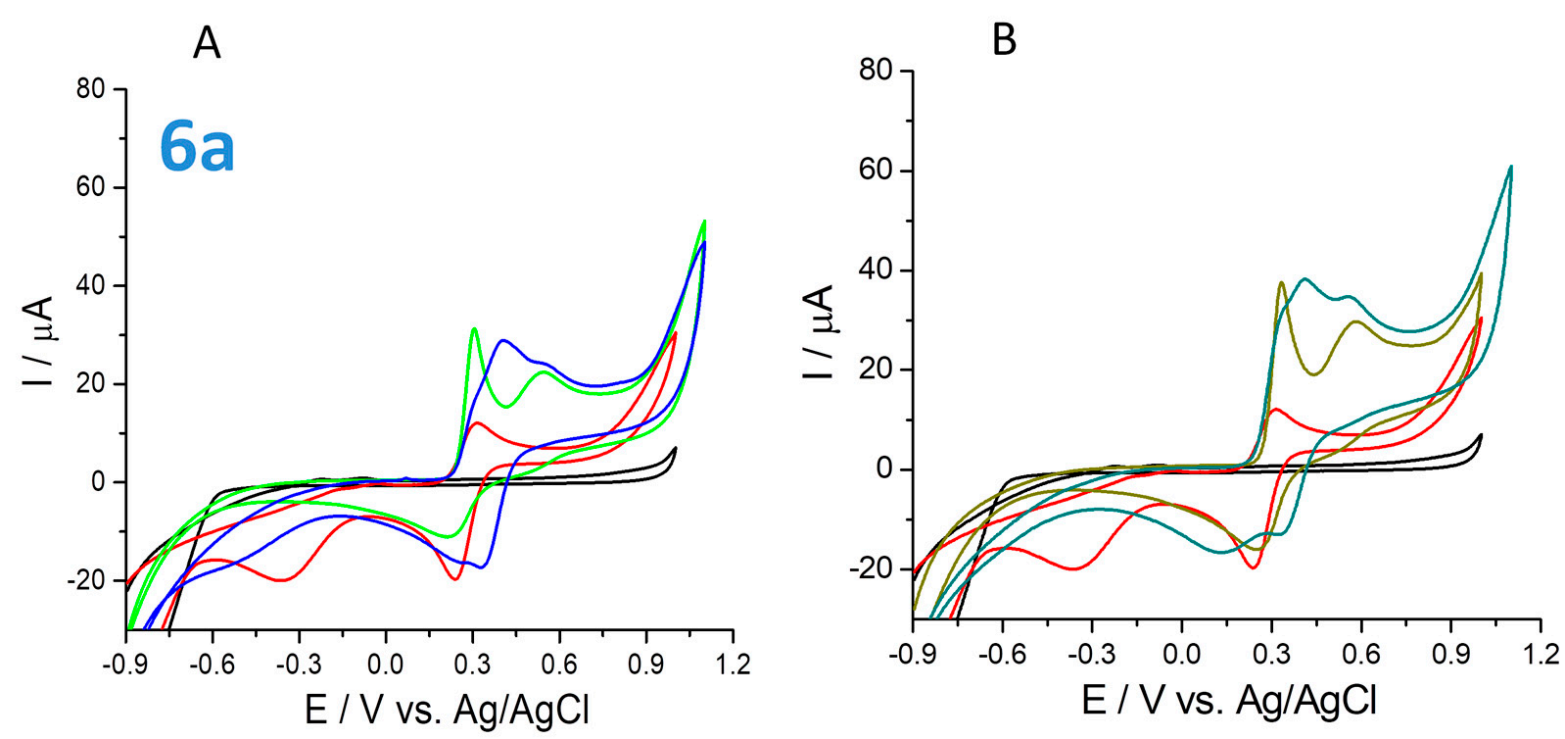
Cyclic voltammograms obtained using graphite-based SPEs in: 2 mM TEMPO prepared using 0.1 M HClO_4_ (red) compared with: (**A**) **6a** 250 μM in HClO_4_ (green); TEMPO 2 mM + **6a** 250 μM after a 60 min reaction conducted in the absence of light (blue); (**B**) **6a** 500 μM in HClO_4_ (dark yellow); TEMPO 2 mM + **6a** 500 μM after a 60 min reaction conducted in the absence of light (dark cyan). The voltammogram of 0.1 M HClO_4_ electrolyte is depicted using black color.

**Figure 12:**
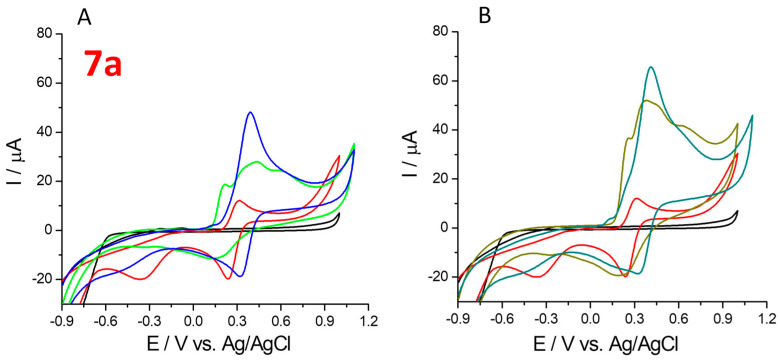
Cyclic voltammograms obtained using graphite-based SPEs in: 2 mM TEMPO prepared using 0.1 M HClO_4_ (red) compared with: (**A**) **7a** 250 μM in HClO_4_ (green); TEMPO 2 mM + **7a** 250 μM after a 60 min reaction conducted in the absence of light (blue); (**B**) **7a** 500 μM in HClO_4_ (dark yellow); TEMPO 2 mM + **7a** 500 μM after a 60 min reaction conducted in the absence of light (dark cyan). The voltammogram of 0.1 M HClO_4_ electrolyte is depicted using black color.

**Figure 13:**
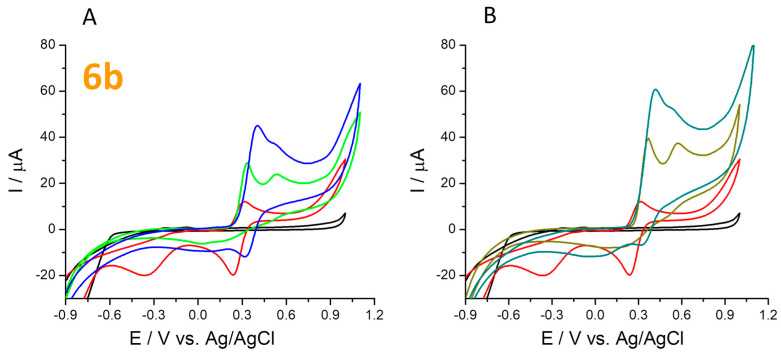
Cyclic voltammograms obtained using graphite-based SPEs in: 2 mM TEMPO prepared using 0.1 M HClO_4_ (red) compared with: (**A**) **6b** 250 μM in HClO_4_ (green); TEMPO 2 mM + **6b** 250 μM after a 60 min reaction conducted in the absence of light (blue); (**B**) **6b** 500 μM in HClO_4_ (dark yellow); TEMPO 2 mM + **6b** 500 μM after a 60 min reaction conducted in the absence of light (dark cyan). The voltammogram of 0.1 M HClO_4_ electrolyte is depicted using black color.

**Figure 14:**
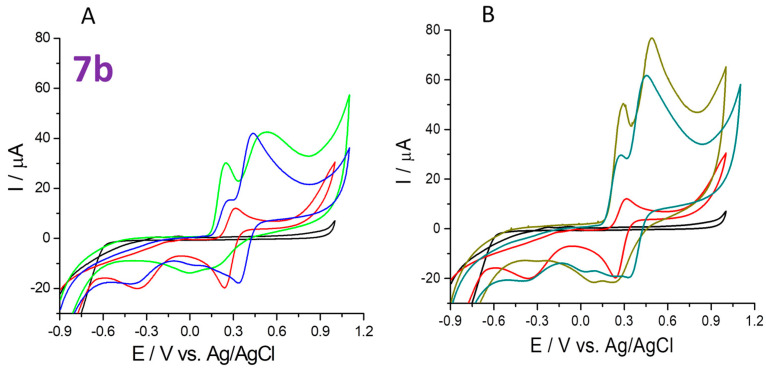
Cyclic voltammograms obtained using graphite-based SPEs in: 2 mM TEMPO prepared using 0.1 M HClO_4_ (red) compared with: (**A**) **7b** 250 μM in HClO_4_ (green); TEMPO 2 mM + **7b** 250 μM after a 60 min reaction conducted in the absence of light (blue); (**B**) **7b** 500 μM in HClO_4_ (dark yellow); TEMPO 2 mM + **7b** 500 μM after a 60 min reaction conducted in the absence of light (dark cyan). The voltammogram of 0.1 M HClO_4_ electrolyte is depicted using black color.

The overlapped voltammograms obtained in the presence of 2 mM TEMPO and 2 mM TEMPO contacted for 60 min in the dark with 250 μM (A) and 500 μM (B), respectively, of the novel compounds are presented in Figure 11, Figure 12, Figure 13 and Figure 14. 

Table 15 shows only the values of the oxidation and reduction potentials and currents corresponding to TEMPO electrochemical transformation at the graphite electrode, in order to observe the comparative scavenging activity of the four analytes and to classify them from this point of view.

It can be observed from Table 15 that all four compounds caused the reduction in the oxidation peak of TEMPO but having the compound signal in the same potential window with it makes it difficult to establish an order. After studying the shape of the voltammograms, obtained for the solutions resulting from the compounds and the interaction with TEMPO and the voltammograms of analytes alone in the solution, it can be concluded that compounds **7b** and **7a** would have the most accentuated antioxidant properties.

### 2.5. Cytotoxicity of the Compounds

**Figure 15:**
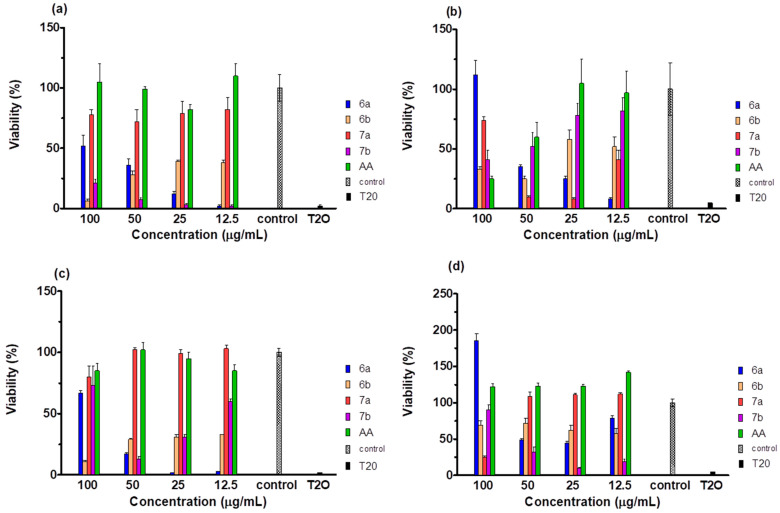
Cell viability determined through the MTT assay: (**a**)—HaCaT cells, (**b**)—BJ cells, (**c**)—A375 cells and (**d**)—A549 cells.

All types of cells reacted differently to each compound (Figure 15). Compound **6a** had a proliferative effect against normal fibroblasts (BJ) and lung adenocarcinoma (A549) cell lines at 100 µg/mL concentration but had inhibitory effects against normal keratinocytes (HaCaT) and skin melanoma (A375) at the same concentration. Compound **7b** had a dose-dependent effect against all cell lines, but with different correlations depending on the type of cells. Against the A375 melanoma and BJ cells, the inhibition was high at higher concentrations, while for the A549 adenocarcinoma and HaCaT cells, the inhibition was high at lower concentrations.

The half-maximal inhibitory concentration (IC_50_) was determined using the 3-(4,5-dimethylthiazol-2-yl)-2,5-diphenyltetrazolium bromide (MTT) method (Table 16). HaCaT cell lines were more sensitive to **6b** and **7b** with IC_50_ values lower than those for **6a** and **7a**; BJ cell lines were more sensitive to **7a** and **7b**, compared to **6a** and **6b**; A375 reacted similarly to HaCaT, while A549 was most sensitive to **6a** and most resistant to **6b**.

**Figure 16:**
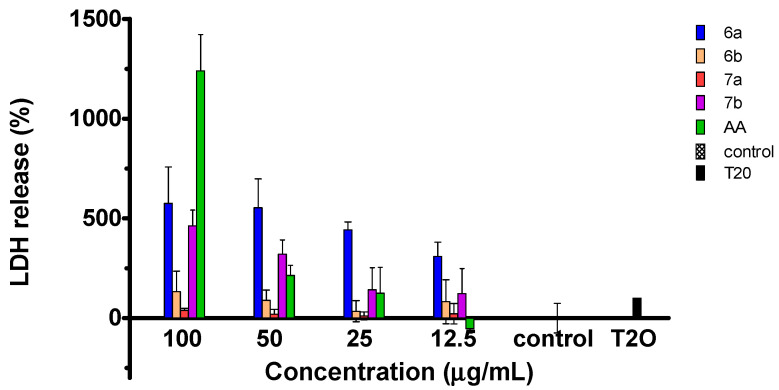
LDH release for HaCaT cell line treated with compounds **6a–b** and **7a–b**.

Ascorbic acid favored the lactate dehydrogenase (LDH) release in HaCaT cells with extremely high values (Figure 16). Otherwise, for the rest of the compounds used, the results showed high levels of LDH in the media at high concentrations.

**Figure 17:**
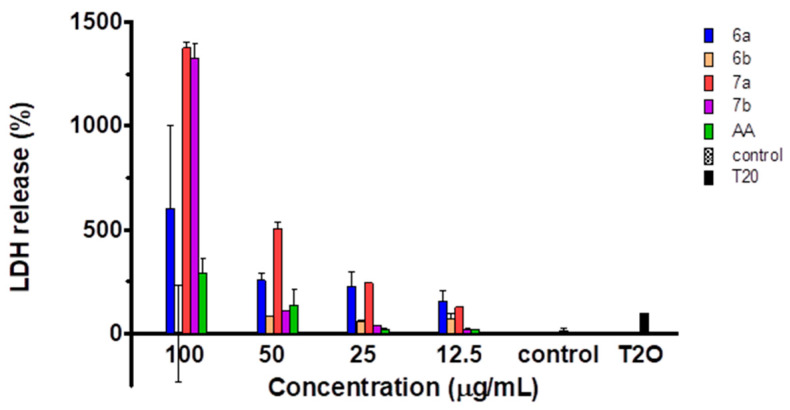
LDH release for BJ cell line treated with compounds **6a–b** and **7a–b**.

For the BJ cell lines, high levels of LDH release were observed for **7a** and **7b** (Figure 17). These results could be correlated to the observed effects during the viability assay, where the same two compounds had inhibitory effects against BJ cells.

**Figure 18:**
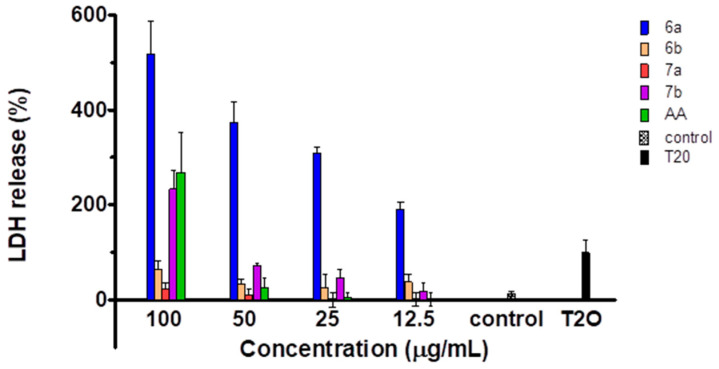
LDH release for A375 cell line treated with compounds **6a–b** and **7a–b**.

The A375 cells had a similar response to the normal cell lines, regarding the LDH release (Figure 18). As observed during the viability assay, **6a** was the most toxic compound for this cell line, and the LDH release was highest for this compound, compared to the other treatments.

**Figure 19:**
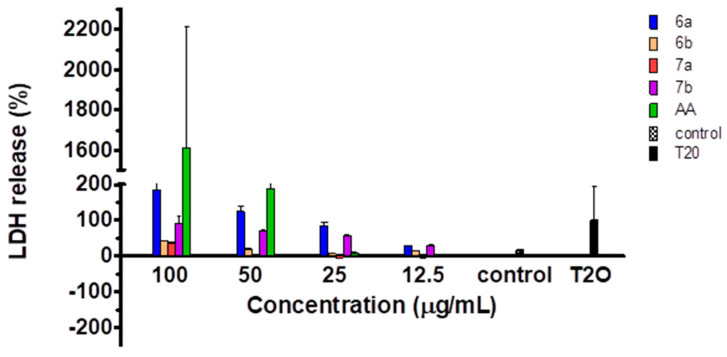
LDH release for A549 cell line treated with compounds **6a–b** and **7a–b**.

The A549 cell line had a similar response to HaCaT when treated with ascorbic acid (Figure 19). Nevertheless, high levels of LDH were observed for **6a** and **7b**, similar to HaCaT, which indicate that these two compounds had the most damaging effects against these two types of cells. 

**Figure 20:**
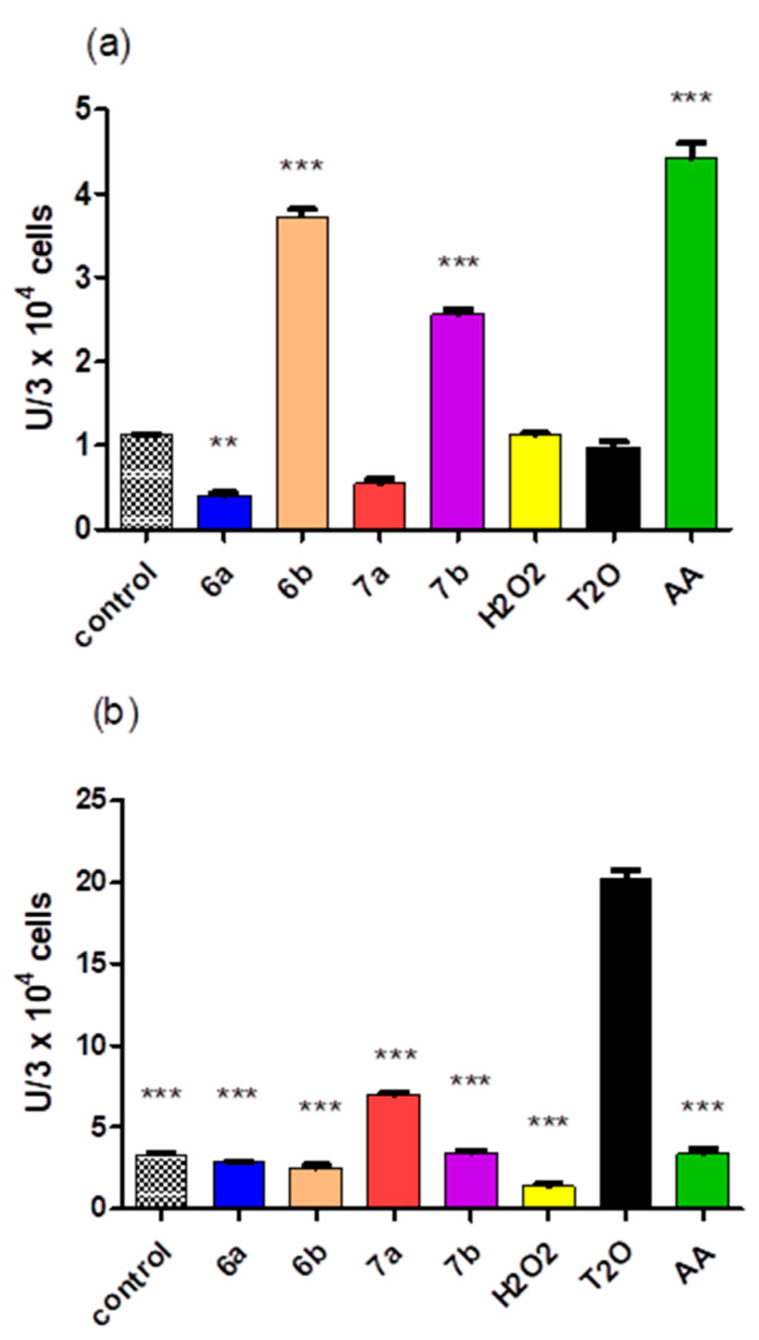

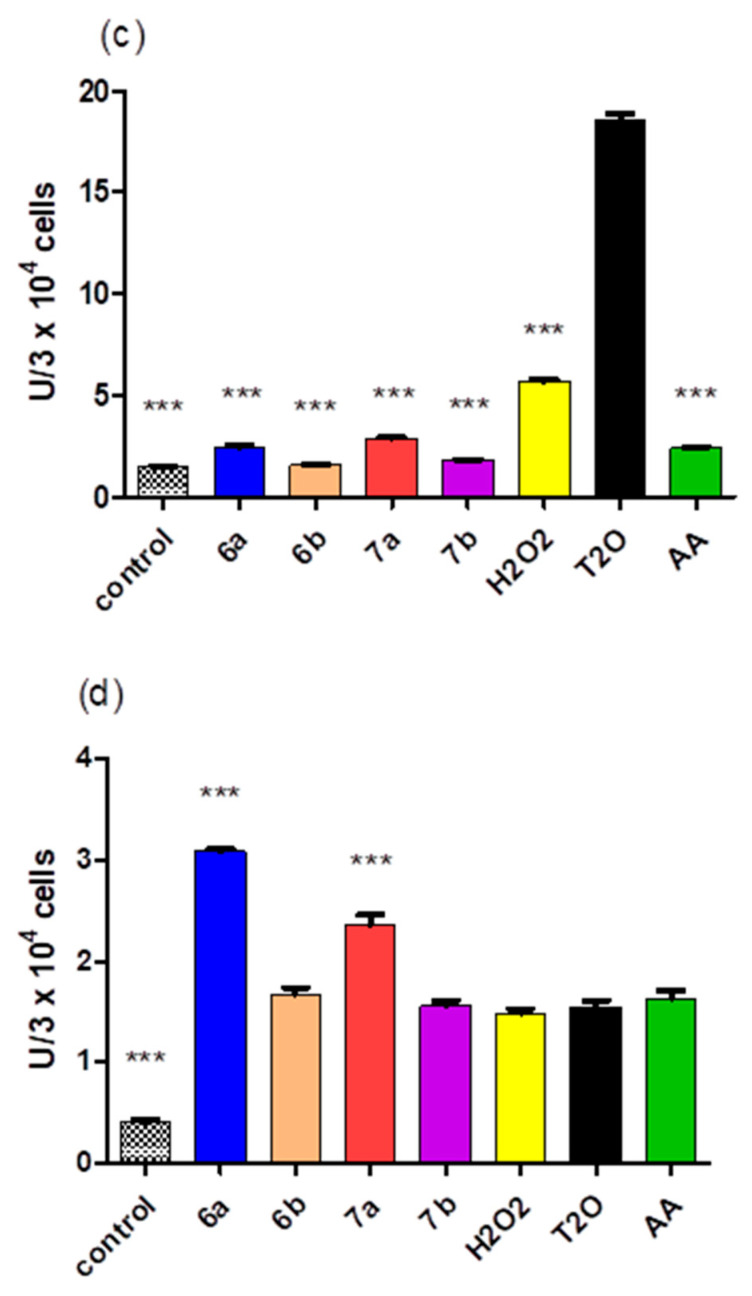
The peroxidase activity in the cell lysates: (**a**) in the A375 cells. The pro-oxidant action of **6b**, **7b** and **AA** (*p* < 0.001) was compared to T2O experimental group, whereas the exposure to **6a** compound had significant antioxidant activity (*p* < 0.01), compared to the reference condition (T2O). (**b**) in the A549 cell type. The enzyme activity was significantly (*p* < 0.001) decreased in all experimental groups, as compared to T2O. (**c**) in the BJ cell line. The T2O treatment on the BJ cell line had a significant (*p* < 0.001) effect in reducing the peroxidase activity in all the other groups. (**d**) in the HaCaT cell line, the peroxidase activity decreased significantly in the control group (*p* < 0.001) and had an opposite effect on the **6a** and **7a** groups (*p* < 0.001). ** *p* < 0.01 and *** *p* < 0.001 when comparisons were made with the T2O group.

**Figure 21:**
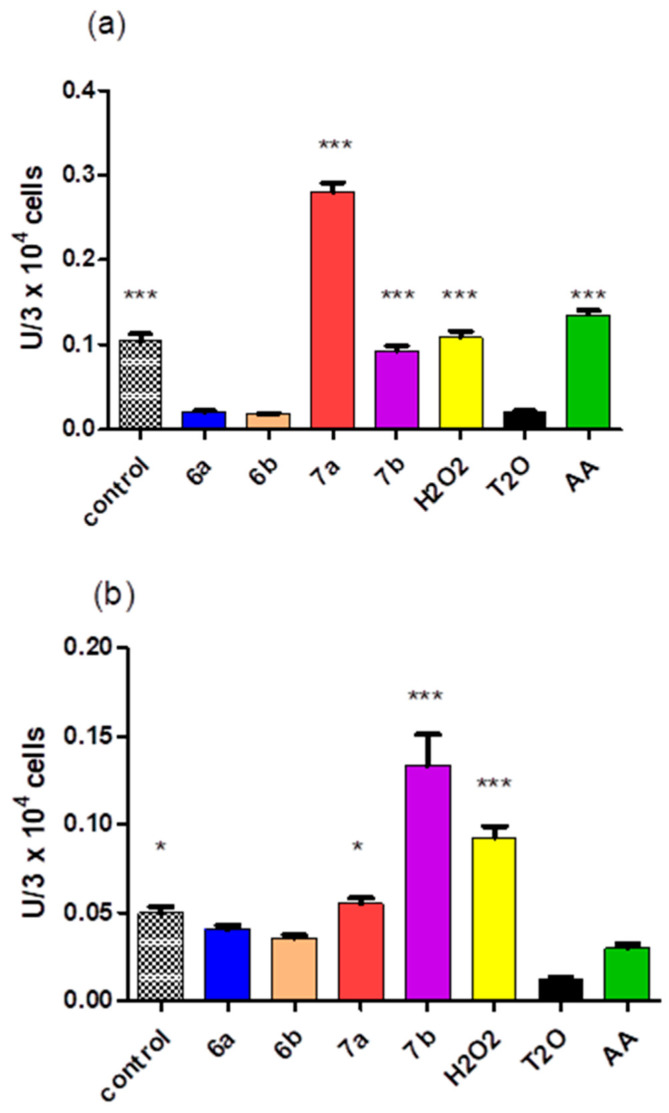

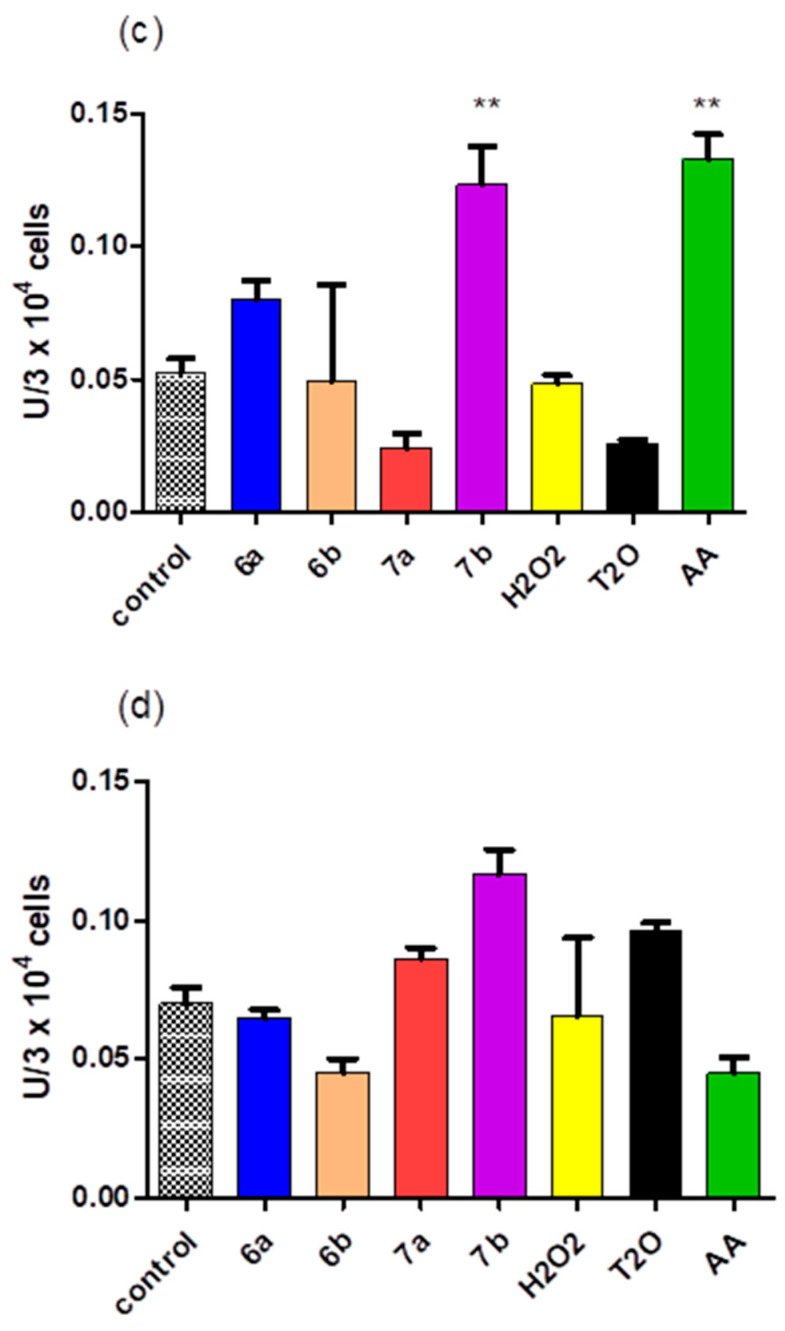
The catalase activity in the studied cell lysates: (**a**) in the A375 cells. In comparison with T2O treatment, in control group as well as **7a**, **7b**, H_2_O_2_ and AA, the catalase activity was significantly increased (*p* < 0.001). (**b**) in the A549 cell lysates exposed to different treatments. Compared to T2O group (Tween 20), the enzyme activity was increased in control group and **7a** (*p* < 0.05) and also significantly increased in **7b** and H_2_O_2_ groups (*p* < 0.001). (**c**) in the BJ cell line. Compared to the T2O experimental group, the enhanced activity of the catalase was noticed only in the **7b** and AA groups (*p* < 0.01). (**d**) in HaCaT cells. The treatments applied on the cells showed no significant effects on hydrogen peroxide production, and then catalase activity showed no treatment-related changes compared to other cell types. * *p* < 0.05, ** *p* < 0.01 and *** *p* < 0.001 when comparisons were performed with the T2O group.

After the exposure of the A375 cell line to the treatments, the oxidative stress markers (peroxidase and catalase activity) varied independently of the type of the treatment. As compared to the group where cells were exposed to Tween 20 (T2O group), the peroxidase activity (Figure 20) increased for **6b**, **7b** and the ascorbic acid group (*p* < 0.001). In turn, catalase activity (Figure 21) increased in the control group, **7a**, **7b**, H_2_O_2_ and the ascorbic acid group (*p* < 0.001). The antioxidant effect of the **6a** treatment was noticed by significantly lowering the peroxidase activity (*p* < 0.05), as compared to the polysorbate-treated cells. The treatment applied to the A549 cell line reduced the peroxidase activity significantly on all the other treatments, compared to T2O group (*p* < 0.001). The same A549 cell line responded differently regarding the catalase activity, where, compared to the polysorbate treatment, increased in the control group, **7a** (*p* < 0.01) and significantly increased in **7b** and the H_2_O_2_ groups (*p* < 0.001). The same T2O treatment on the BJ cell line had a similar effect in reducing the peroxidase activity of all the other groups, compared to T2O (*p* < 0.001), whereas the catalase activity increased in **7b** and the ascorbic acid groups (*p* < 0.05). The peroxidase activity on the HaCaT cell line decreased significantly in the control group (*p* < 0.001) and had an opposite effect on the **6a** and **7a** groups (*p* < 0.001), as compared to T2O. From the perspective of catalase activity, the polysorbate 20 (T2O) treatment showed no effect in increasing or decreasing the catalase activity of all the other groups.

The enzyme’s activity was measured in accordance with the protein concentration (0.038 ± 0.002 mg/mL in A375 cells, 0.03 ± 0.001 mg/mL in HaCaT cells, 0.034 ± 0.0023 mg/mL in A549 cells and 0.05 ± 0.003 mg/mL in BJ cells), calculated with molar extinction coefficient 43.8 M^−1^ cm^−1^ and then reported to the cells number (3 × 10^4^ cells/group).

## 3. Materials and Methods

### 3.1. Validation of the Design Hypothesis Using In Silico Thermodynamic Calculations

The Bond Dissociation Enthalpy (BDE) is a key descriptor used for the characterization of the antioxidant activity of phenolic compounds. It assesses the homolytic break of the O-H bond, releasing a hydrogen atom (proton + electron simultaneously), corresponding to the hydrogen atom transfer (HAT) mechanism. In the field of phenolic antioxidants, the single electron transfer-proton transfer (SET-PT) and the sequential proton loss electron transfer (SPLET) mechanisms are not the best mechanisms through which phenols act as antioxidants [24,25,26]. The release of hydrogen atoms (hydrogen atom transfer) can be presented in a simplified way as follows: antioxidant-H + radical^•^ −> antioxidant^•^ + radical-H.

In the field of development and evaluation of new antioxidant agents, the connection with the theoretical in silico calculations was identified. Many groups of researchers have identified a link between the easiness of a molecule’s hydrogen atom release from computational work with the results of the in vitro experiments [25,27,28,29,30,31]. 

The facility that characterizes a heterolytic cleavage of a covalent bond is strongly connected to the capacity of the resulting radical to stabilize and have the lowest possible energetic state, thanks to internal conjugation. The internal conjugation of a radical can be largely influenced by the chemical structure of the studied compound (substituents, position of the substituents, internal hydrogen bonding, conformation, etc.) [11,31].

The experience of our group is mainly in the field of polyphenolic agents, where the in silico thermodynamic calculations provided the data with good correlations regarding the in vitro activity of the compounds [16,18,19]. The calculation of the BDE was performed in silico, according to a previously reported protocol [12].

In our previous report we identified that there is a possible influence of the substituents of the thiazole ring on the easiness of N-H and O-H homolytic breaking, with consequences on the antioxidant activity of the compounds [3]. The aim of the present study was to obtain catechol-derived compounds, based on the fact that they exhibit higher activity compared to the non-catechol polyphenols. Thus, a library of theoretical compounds was created, using various substituents in positions 4 and 5 of the thiazole and with a catechol moiety on the benzylidene fragment. The choice of those types of substituents was to include a large area of the types of electronic and steric effects, such as electron donating groups, electron withdrawing groups, small groups or bulky groups. The types of substituents were selected according to the commercial availability of the α-halo carbonyl reagents available from suppliers of chemical reagents.

### 3.2. Chemical Synthesis

**Scheme 1:**
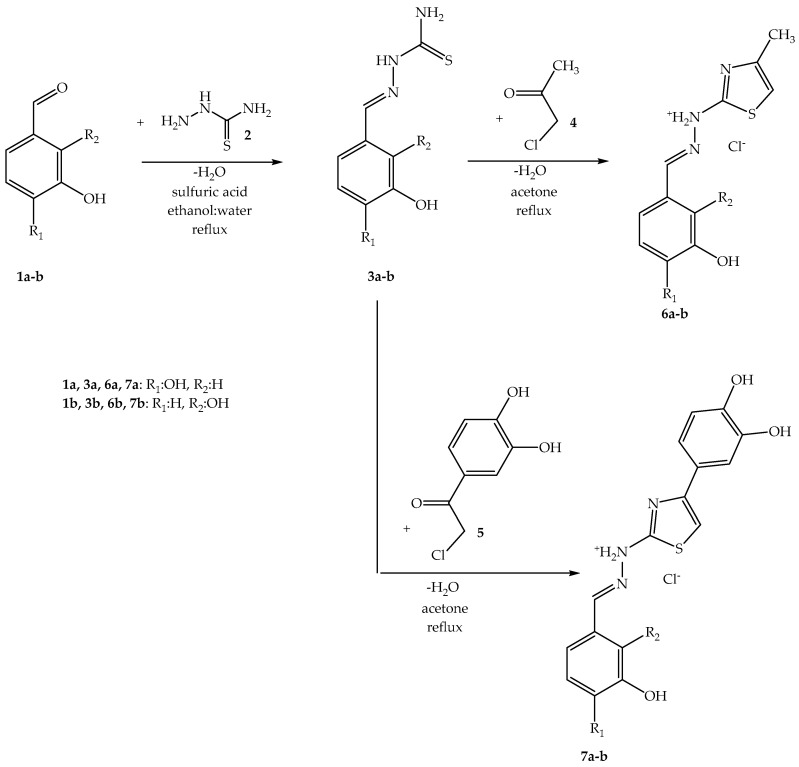
General synthetic route for compounds **6a–b** and **7a–b**.

All chemicals and consumables used in the research were purchased from local suppliers. The route of synthesis of the intermediate thiosemicarbazones **3a–b** and the final compounds **6a–b** and **7a–b** is illustrated in Scheme 1.

The synthesis of the intermediate thiosemicarbazones **3a–b** was conducted using previously reported protocols [32], which were adapted to current research. The synthesis consisted in the condensation of the catechol derived benzaldehydes **1a–b** and thiosemicarbazide (2), resulting in the precipitation of thiosemicarbazones **3a–b** from the reaction environment. The obtention of thiosemicarbazone **3a** was previously reported by our group, and the same protocol was applied for the obtention of thiosemicarbazone **3b** [10]. The physical and spectral characterization of the intermediate compound **3b** confirmed its successful obtention, being in agreement with the previous reports from the literature [13,33].

The final compounds **6a–b** and **7a–b** were synthesized by reflux heating, using a 5 mmol water bath of the intermediate thiosemicarbazones **3a–b** with 5.05 mmol of α-haloketones 4 and 5 in 30 mL of anhydrous acetone for one hour. After cooling the reaction mixture, the resulting precipitate was filtered under vacuum and later crystallized twice from acetone (2 × 50 mL). The intermediate compound **3a** and the final compound **6a** were reported before in a paper by our group, being the starting point for research in the current paper [10].

The numerical values corresponding to the peaks from the spectra recorded for the compounds from the present paper are presented below. The compounds’ spectra are depicted in the Supplementary Materials (Figures S1–S14), along with the highest occupied molecular orbital (HOMO) and lowest unoccupied molecular orbital (LUMO) of compounds **6a–b** and **7a–b** (Table S1) and their electrostatic potential maps (Table S2).

(E)-2-(2,3-dihydroxybenzylidene)-1-(4-methylthiazol-2-yl)hydrazin-1-ium chloride (**6b**): yellow solid; carbonization over 240 °C; yield = 51%; FT IR (KBr) ν_max_ cm^−1^: 3548, 3474, 3412, 3293, 3229, 1628, 1620, 1580, 1559, 1513, 1501, 1476, 1364, 1335, 1278, 1247, 1158, 1109, 988; MS: *m/z* = 248.2 (M − 1); ^1^H-NMR (dimethyl sulfoxide-*d6* (DMSO-*d_6_)*, 500 MHz) δ: 2.226 (s, 3H, -CH_3_), 6.617 (s, 1H, ThC5), 6.711 (t, 1H, Ar, *J* = 8 Hz), 6.879 (dd, 1H, Ar, *J* = 7.5 Hz and *J* = 1.5 Hz), 7.188 (d, 1H, Ar, *J* = 7.5 Hz), 8.601 (s, 1H, -CH=N); ^13^C-NMR (DMSO-*d_6_*, 125 MHz) δ: 14.404 (-CH_3_), 103.098 (ThC5), 117.174 (Ar), 117.531 (Ar), 119.526 (Ar), 119.890 (Ar), 139.089 (ThC4), 145.913 (Ar-OH), 145.857 (-CH=N), 146.438 (Ar-OH), 167.317 (ThC2).

(E)-2-(2,3-dihydroxybenzylidene)hydrazine-1-carbothioamide (**3b**): pale yellowish solid; mp = 206 °C [lit. 206–207 °C [33], 479–581 K [13]); yield = 81%; FT IR (KBr) ν_max_ cm^−1^: 3336 (OH), 3291 (N-H), 3257, 3175, 2831, 1620 (C=N), 1611, 1601, 1580, 1538, 1507, 1474, 1358, 1340, 1279, 1248, 1199, 1159, 1104, 1068, 982; MS: *m/z* = 210.0 (M − 1);

(E)-2-(3,4-dihydroxybenzylidene)-1-(4-(3,4-dihydroxyphenyl)thiazol-2-yl)hydrazin-1-ium chloride (**7a**): yellow solid; carbonization over 230 °C; yield = 48%; FT IR (KBr) ν_max_ cm^−1^: 3288 (OH), 3123 (N-H), 1619 (C=N), 1523, 1511, 1465, 1330, 1290, 1285, 1198, 1166, 1118, 1099; MS: *m/z* = 342.3 (M − 1); ^1^H-NMR (DMSO-*d_6_*, 500 MHz) δ: 6.792 (m, 2H, Ar), 6.919 (d, 1H, Ar, *J* = 8.5 Hz), 6.967 (s, 1H, ThC5), 7.097 (d, 1H, Ar, *J* = 8.5 Hz), 7.174 (s, 1H, Ar), 7.204 (s, 1H, Ar), 8.008 (s, 1H, -CH=N); ^13^C-NMR (DMSO-*d_6_*, 125 MHz) δ: 100.789 (ThC5), 112.638 (Ar), 113.604 (Ar), 115.858 (Ar), 117.496 (Ar), 120.254 (Ar), 124.628 (Ar), 125.524 (Ar), 144.891 (Ar-OH), 145.402 (Ar-OH), 145.843 (-CH=N), 145.934 (Ar-OH), 147.917 (Ar-OH), 168.297 (ThC2).

(E)-2-(2,3-dihydroxybenzylidene)-1-(4-(3,4-dihydroxyphenyl)thiazol-2-yl)hydrazin-1-ium chloride (7b): yellow solid; carbonization over 250 °C; yield = 54%; FT IR (KBr) ν_max_ cm^−1^: 3410 (OH), 3229, 3115 (N-H), 2957, 2924, 2851, 2832, 1614 (C=N), 1579, 1469, 1364, 1320, 1284, 1264, 1246, 1209, 1177, 1129, 972, 771, 734, 595; MS: *m/z* = 342.4 (M − 1); ^1^H-NMR (DMSO-*d_6_*, 500 MHz) δ: 6.713 (t, 1H, Ar, *J* = 8 Hz), 6.773 (d, 1H, Ar, *J* = 8 Hz), 6.826 (dd, 1H, Ar, *J* = 8 Hz and *J* = 1.5 Hz), 6.965 (s, 1H, ThC5), 7.066 (dd, 1H, Ar, *J* = 7.75 Hz and *J* = 1 Hz), 7.111 (dd, 1H, Ar, *J* = 8 Hz and *J* = 2 Hz), 7.220 (d, 1H, Ar, *J* = 2 Hz), 8.381 (s, 1H, -CH=N); ^13^C-NMR (DMSO-*d_6_*, 125 MHz) δ: 100.537 (ThC5), 113.555 (Ar), 115.837 (Ar), 116.698 (Ar), 117.391 (Ar), 117.517 (Ar), 119.547 (Ar), 120.324 (Ar), 125.328 (Ar), 142.455 (Ar-OH), 145.059 (Ar-OH), 145.353 (-CH=N), 145.738 (Ar-OH), 149.028 (Ar-OH), 167.786 (ThC2).

The melting points of the synthesized compounds were determined using the glass capillary method in a MPM-H1 melting point device (Schorpp Gerätetechnik, Überlingen, Germany). The infrared (IR) spectra of the compounds were recorded using a FT/IR 6100 spectrometer (Jasco, Cremella, Italy) in KBr pellets. The mass spectra of the compounds were recorded in negative ionization mode on an Agilent 1100 series device, connected to an Agilent Ion Trap SL mass spectrometer (Agilent Technologies, Santa Clara, CA, USA). The proton nuclear magnetic resonance (^1^H-NMR) and carbon nuclear magnetic resonance (^13^C-NMR) spectra of the compounds were recorded after their dissolution in dimethyl sulfoxide-*d_6_* (DMSO-*d_6_*), using an Avance NMR spectrometer (Bruker, Karlsruhe, Germany). Tetramethylsilane (TMS) was used for calibrating the NMR spectrometer and the peak of the solvent was used as a reference for calculating the chemical shift values.

### 3.3. In Vitro Antiradical, Electron Transfer and Metal Ions Chelation Assays

The evaluation of the antioxidant capacity of the compounds was performed based on the different possible mechanisms reported in the literature, scavenging external radicals, electron transfer or by the chelation of some transition metal ions. The solid powders of the tested compounds and reference compounds were dissolved in DMSO from a newly opened bottle to obtain 2 mM starting solutions, which were further diluted using DMSO according to specific needs of each assay. All absorbance measurements were performed using an UV-VIS Jasco V-530 spectrophotometer (Jasco International Co., Tokyo, Japan) at room temperature against specific blank samples for each assay and with a spectral resolution of 1 nm. Prior to performing absorbance measurements in the assays, the recorded spectra of the tested compounds indicated no maximum absorbance near the wavelengths where the measurements were needed to be carried out for each assay. Results are presented as averages of three determinations.

The protocols followed for performing all assays in the current paper were already reported by our group in our previous papers [17].

#### 3.3.1. Antiradical Assays

The assay of scavenging 1,1-diphenyl-2-picrylhydrazyl (DPPH^•^) consists of the transfer of a hydrogen atom from the studied substance to the strong-colored radical due to its odd electron, with an absorption maximum at λ = 517 nm. The scavenging of the DPPH^•^, due to the absorbance lowering of the assay mixture solutions, was calculated using Equation (1).

(1) $$radical\;scavenging\;(\%)=\frac{control\;absorbance-sample\;absorbance}{control\;absorbance}\times 100$$

The assay of scavenging 2,2′-azino-bis(3-ethylbenzothiazoline-6-sulfonic acid) (ABTS^•+^) was performed in potassium phosphate buffer (0.1 M, pH = 7.4) after activation of the reagent with MnO_2_. The decrease in the absorbance at λ = 734 nm of the assay mixture solutions was determined spectrophotometrically, and the percent of radical scavenged was calculated using Equation (1). 

The assay of singlet molecular oxygen (^1^O_2_) scavenging was carried out using an indirect method, previously reported by our research team [10]. Briefly, indocyanine green (ICG), purchased from Sigma–Aldrich (St. Louis, MO, USA), was used to generate ^1^O_2_ in the presence of compounds **6a**, **6b**, **7a** and **7b** and alone as a reference, while exposed to a 785 nm laser diode (Micro Raman System R-3000, 190 mW power) for 60 s. To determine the ^1^O_2_ scavenging capacity of the studied molecules, we have monitored the degradation of 1,3-diphenylisobenzofuran (DPBF), procured from Alfa Aesar (Haverhill, MA, USA), using an UV-VIS spectrophotometer from Jasco International Co., Ltd. (Japan), model V-530. The final concentrations used in this experiment were 0.1 mM for DPBF, 10^−5^ M for ICG and 10^−6^ M for the ^1^O_2_ scavengers. Free DPBF was used as control.

#### 3.3.2. Electron Transfer Assays

All electron transfer assays were performed using equimolecular quantities of analyzed compounds and reference compounds, evaluated in the same experimental conditions in each assay [16,17]. The activity of a compound, which referred to the activity of the equimolecular amount of a reference compound (trolox or ascorbic acid), was calculated using Equation (2).

(2) $$\%\;of\;control\;activity=\frac{sample\;absorbance}{reference\;absorbance}\times 100$$

The Ferric-Reducing Antioxidant Potential (FRAP) assay is based on the initial report of Benzie and Strain with some adaptations [34]. The amount of Fe^2+^, resulting from the reduction of Fe^3+^ from the reagent due to the redox activity of the analyzed compound, was measured by complexation with a chromogenic ligand, providing a strong blue complex with an absorption maximum at λ = 593 nm. 

The Total Antioxidant Capacity (TAC) was determined using the reduction of the phosphomolybdate reagent by the tested compounds at heating, providing a green complex with an absorption maximum at λ = 695 nm. 

The Reducing Power (RP) assay contributed to the reduction of the [Fe(CN)_6_]^3−^ to [Fe(CN)_6_]^4−^ due to the transfer of an electron from the analyzed compound. The amount of ferrocyanide provides a blue complex in the presence of ferric ions, with an absorption maximum at λ = 700 nm. 

#### 3.3.3. Metal Ions Chelation Assays

The assessment of the chelation capacity of the synthesized compounds to the ferrous ions was based on their competition with ferrozine for the metallic ions. The spectrophotometric measurements were made at λ = 562 nm, corresponding to the absorbance of the red-colored complex formed by ferrozine with ferrous ions. The presence in samples of a chelator agent results in a decrease of color due to the disruption of the ferrozine–ferrous ions complex. Ethylenediaminetetraacetic acid disodium salt (EDTA-Na_2_) was used as the reference compound.

Evaluation of the cupric ions chelation capacity was conducted using murexide as the chromogenic chelator, and measurements were made at 485 nm and 520 nm, corresponding to the absorbance of murexide-copper(II) complex and free murexide, respectively. The ratio of the two absorbances is relative to the amount of free copper(II) in the solution. EDTA-Na_2_ was used as the reference compound. The cupric ions chelation capacity of the compounds was determined using Equation (3):

(3) $$copper\;chelation\left(\%\right)=\frac{{\left(\frac{A_{485}}{A_{520}}\right)}_{control}-{\left(\frac{A_{485}}{A_{520}}\right)}_{sample}}{{\left(\frac{A_{485}}{A_{520}}\right)}_{control}}\times 100$$

### 3.4. Electrochemical Behavior of Compounds

The stock solutions of the used synthesized compounds and references were prepared as 5 mM solutions in dimethylformamide (DMF). To test their redox properties, dilutions at 250, 500 and 1000 μM were prepared in different electrolytes such as 0.1 M HClO_4_ with pH = 1.2, 0.1 M acetate buffer (AB) with pH = 4 and 40 mM of Britton–Robinson buffer (BRB) with pH = 4. Cyclic voltammetry tests were performed using screen-printed electrodes (SPEs) based on carbon and gold, provided by Metrohm Dropsens Spain. Different experimental parameters (optimized) were used depending on the type of test and the nature of the electrochemical process followed in each test.

The possible antioxidant properties were investigated in terms of their activity, such as:

1. ferric ions (Fe^3+^) reducing antioxidant power (cyclic voltammetry by scanning the potential range between −0.5 V to 1 V with a scan rate of 100 mV s^−1^);
2. hydrogen peroxide (H_2_O_2_) scavenging (cyclic voltammetry by scanning the potential range between −0.2 V to 0.7 V with a scan rate of 100 mV s^−1^);
3. the 1,1-diphenyl-2-picrylhydrazyl (DPPH^•^) free radical scavenging (cyclic voltammetry test in the potential range of −0.5 to 1 V after 30 min, and 120 min reaction of each compound and DPPH^•^ at 20 °C room temperature in the dark);
4. 2,2,6,6-tetramethylpiperidinyl-1-oxy (TEMPO) scavenging (cyclic voltammetry test in the potential range of −1 to +1 V after 60 min reaction of each compound and TEMPO at 20 °C room temperature in the dark).

### 3.5. Cytotoxicity of the Compounds

The cytotoxicity of compounds **6a–b** and **7a–b** was studied against four cell lines: normal keratinocytes (HaCaT), normal fibroblasts (BJ), skin melanoma (A375) and lung adenocarcinoma (A549). The cell viability and consequent membrane integrity were assessed through 3-(4,5-dimethylthiazol-2-yl)-2,5-diphenyltetrazolium bromide (MTT) and lactate dehydrogenase (LDH) assays, respectively. The methodologies used are described in a previous paper [35]. Briefly, cell lines were left to attach to the 96 wells plate for 24 h at different confluences (10^4^ cancerous cell lines and 12 × 10^3^ normal cell lines). The attached cells were exposed to the tested compounds for 24 h at different concentrations (from 12.5 to 100 µg/mL). After this interval, the media were assessed for the LDH assay, and the cell viability was determined through the MTT assay. Based on the MTT assay, the concentration, at which 50% of cells were affected (IC_50_), was calculated.

The IC_50_ concentration was used for further tests to determine the activity of catalase and peroxidase, as markers of cell response to the oxidative stress. The cells were left to attach on 12 wells plates for 24 h and then they were exposed to the tested compounds for another 24 h. Cellular lysates were made using polysorbate 20 (Tween 20) and were then examined for the two enzymes’ activity determination.

Peroxidase activity was measured using a 50 mM citrate-disodium phosphate buffer, pH = 5, with a concentration of 16.6 mg/100 mL ortho-dianisidine dihydrochloride produced by Sigma–Aldrich (St. Louis, MO, USA) in citrate buffer, according to a previously reported method [36]. The absorbance, at 405 nm using 50 µL of cell lysate and 2950 μL buffer, was measured spectrophotometrically every 10 s for 2 min. The activity of peroxidase measured was calculated using the standard curve of peroxidase activity, ranging from 1–5 U/mL, and was expressed according to the number of cells from the cell lysate. The catalase activity was measured using a standard buffer, the activity at 240 nm was identified, and calculated accordingly [37].

## 4. Conclusions

Several catechol derivatives with potentially enhanced antioxidant properties were designed, starting from a previously reported compound with good antioxidant properties (2-(3,4-dihydroxybenzylidene)-1-(4-methylthiazol-2-yl)hydrazin-1-ium chloride (CHT)) and based on in silico thermodynamic calculations. After the chemical synthesis, the intermediate and final compounds were physicochemically characterized. Their antioxidant activity was assessed through various antiradical, electron transfer and metal chelation assays, and their electrochemical behavior and cytotoxicity were also studied. 

The results obtained in the in vitro experiments generally correlated very well with the in silico studies, with all final compounds presenting very good antioxidant properties. Overall, the 2,3-dihydroxybenzylidene derivatives (series **b**) exhibited better radical scavenging properties than the 3,4-dihydroxybenzylidene derivatives (series **a**), with all compounds being more active than the reference antioxidants used. Similarly, the compounds from series **b** presented a higher electron donating capacity than those from series **a**, and except compound **6a**, all were superior to the references used. Regarding the ferrous and cupric ions chelating capacity, the compounds from series **b** were more active than those from series **a**.

Generally, the results obtained from studying the compounds’ electrochemical behavior were in good agreement with the results of the in vitro antioxidant activity evaluation assays.

Regarding their cytotoxicity, the studied compounds presented different effects (proliferative or inhibitory), depending on the concentration and cell line used. Compound **7b** had a dose-dependent inhibitory effect against all cell lines. In the MTT assay, HaCaT and A375 cell lines were more sensitive to compounds from series **b** than those from series **a**, whereas the BJ cell line was more sensitive to compounds **7a–b** than to compounds **6a–b**, while A549 was most sensitive to **6a** and most resistant to **6b**. The lactate dehydrogenase (LDH) assay results were in good correlation to those found in the viability assay. Related to the oxidative stress markers, the peroxidase activity was increased by compounds from **b** series in the A375 cell line and was significantly reduced by all compounds in A549 and BJ cell lines. The catalase activity was increased by compounds **7a–b** in the A375 cell line and by **7b** in A549 and BJ cell lines.

In conclusion, the easiness of a molecule’s hydrogen atom release from computational studies correlated well with the results of the in vitro experiments, allowing the design of catechol hydrazones with excellent antioxidant properties. Compound **7b**, which possesses two catechol moieties in its structure, exhibited the best antioxidant activity. 

## Figures and Tables

**Table 1:** BDE (kcal/mol) computed in vacuum regarding the O-H catechol groups and N-H hydrazones of the potential compounds and the influence of substituents on the easiness of hydrogen atoms abstraction from the potential molecules via hydrogen atom transfer mechanism.

Substitution		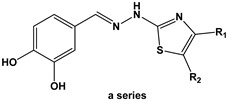			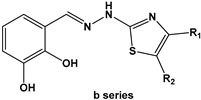		
R_1_	R_2_	O-H BDE * (kcal/mol)	N-H BDE (kcal/mol)	Influence	O-H BDE * (kcal/mol)	N-H BDE (kcal/mol)	Influence
-H	-H	67.70	68.77	medium	62.78	65.12	negative
-CH_3_	-H	67.50	68.71	medium	62.74	65.73	medium
-CH_3_	-CH_3_	67.26	68.04	positive	62.61	71.75	negative
-C(CH_3_)_3_	-H	67.62	68.86	medium	62.78	73.57	negative
-CO-CH_3_	-H	68.38	69.63	negative	62.74	65.55	negative
-COO-CH_3_	-H	68.72	69.72	negative	62.70	65.49	negative
-H	-CO-CH_3_	68.93	70.69	negative	62.66	66.47	negative
-H	-COO-CH_3_	68.70	70.29	negative	62.56	66.06	medium
-C_6_H_5_	-H	67.82	68.83	medium	62.58	64.62	positive
-C_6_H_5_	-C_6_H_5_	67.63	68.38	medium	62.32	71.47	negative
-C_6_H_5_	-CH_3_	67.54	68.33	positive	62.51	71.80	negative
-C_6_H_3_-3,4-diOH	-H	67.68	68.64	medium	62.43	64.19	positive

* Lowest BDE from the two O-H groups of catechol.

**Table 2:** The computed BDEs from the groups that can release hydrogen atoms from the proposed compounds **6a–b** and **7a–b** in vacuum via hydrogen atom transfer mechanism (kcal/mol).

Compound	N-H_1_	O-H_2_	O-H_3_	O-H_4_	O-H_5_	O-H_6_
**6a**	68.71	67.50	70.17			
**6b**	65.73		81.05	62.74		
**7a**	68.64	67.68	70.29		78.45	66.22
**7b**	64.19		81.11	62.43	78.91	67.10

**Table 3:** The computed BDEs from the groups that can release hydrogen atoms from the proposed compounds **6a–b** and **7a–b** in toluene via hydrogen atom transfer mechanism (kcal/mol).

Compound	N-H_1_	O-H_2_	O-H_3_	O-H_4_	O-H_5_	O-H_6_
**6a**	67.99	67.01	69.53			
**6b**	64.46		78.58	62.25		
**7a**	67.85	67.09	69.48		76.35	65.45
**7b**	63.55		78.61	62.10	76.84	66.33

**Table 4:** The computed BDEs from the groups that can release hydrogen atoms from the proposed compounds **6a–b** and **7a–b** in ethanol via hydrogen atom transfer mechanism (kcal/mol).

Compound	N-H_1_	O-H_2_	O-H_3_	O-H_4_	O-H_5_	O-H_6_
**6a**	69.80	66.78	73.29			
**6b**	66.45		79.04	64.98		
**7a**	69.72	66.83	73.31		77.41	69.33
**7b**	65.69		79.12	64.82	77.52	69.82

**Table 5:** The computed BDEs from the groups that can release hydrogen atoms from the proposed compounds **6a–b** and **7a–b** in water via hydrogen atom transfer mechanism (kcal/mol).

Compound	N-H_1_	O-H_2_	O-H_3_	O-H_4_	O-H_5_	O-H_6_
**6a**	69.76	66.25	73.43			
**6b**	66.53		76.98	65.32		
**7a**	69.75	66.34	73.54		77.18	69.43
**7b**	65.69		73.62	65.20	77.34	69.96

**Table 6:** The lowest BDEs of the groups that can release hydrogen atoms computed in vacuum from our group’s previous reports. The lowest BDE presented was for the most active compound in the in vitro assays (kcal/mol).

Paper	Lowest BDE (kcal/mol)	Type of Bond
Marc et al., 2019 [16]	71.56	Phenol O-H
Marc et al., 2021 [17]	62.30	Hydrazone N-H
Pele et al., 2022 [18]	72.00	Phenol O-H
Pele et al., 2022 [19]	71.97	Phenol O-H

**Table 7:** Determined activity of newly catechol derived compounds against DPPH^•^.

Compound	Scavenging Effect (%) at Specific Concentration (µM)											IC_50_ (µM)
	2.5	5	7.5	10	12.5	20	30	50	60	70	80	
6a *	20.40	28.40	37.22	48.79	57.48	81.57	+	+	+	+	+	10.80
**6b**	21.99	33.55	42.15	52.88	60.98	85.12	+	+	+	+	+	9.74
**7a**	35.10	48.08	54.81	66.30	74.31	+	+	+	+	+	+	6.02
**7b**	39.15	51.69	60.89	72.89	81.10	+	+	+	+	+	+	4.85
**Trolox**	−	8.33	13.10	14.67	23.22	31.77	39.67	68.38	83.38	+	+	35.50
**Ascorbic acid**	−	−	−	9.75	12.40	20.04	30.05	46.18	56.35	66.41	73.05	53.39

* Data were previously reported [10]; − depicts < 5%; + depicts > 95%.

**Table 8:** Determined activity of newly catechol derived compounds against ABTS^•+^.

Compound	Scavenging Activity (%) at Given Concentration (µM)								IC_50_ (µM)
	1.5	2.5	3.75	5	7.5	10	17.5	25	
6a *	22.55	28.93	41.32	49.70	70.72	87.20	+	+	5.03
**6b**	24.55	34.74	43.27	58.42	77.85	94.57	+	+	4.37
**7a**	22.15	33.84	58.37	79.67	+	+	+	+	3.28
**7b**	35.16	50.60	64.44	86.46	+	+	+	+	2.55
**Trolox**	−	−	13.10	20.45	25.99	31.72	53.26	77.62	15.90

* Data were previously reported [10]; − represents < 10%; + represents > 95%.

**Table 9:** Results of the assays involving electron transfer (Ferric Reducing Antioxidant Potential (FRAP), Reducing Power (RP) and Total Antioxidant Capacity (TAC)), indicated as a percentage of activity for the newly catechol derived compounds compared to ascorbic acid and trolox.

Compound	% of Activity Compared to Ascorbic Acid			% of Activity Compared to Trolox		
	FRAP	RP	TAC	FRAP	RP	TAC
6a *	201.55	138.54	106.46	171.35	253.86	80.10
**6b**	255.09	223.28	214.42	216.87	409.14	161.33
**7a**	228.88	175.10	185.38	194.58	320.86	139.47
**7b**	312.67	314.45	270.42	265.81	576.19	203.46

* Data were previously reported [10].

**Table 10:** Findings from the assessment of ferrous ions chelation capacity for the newly catechol derived compounds (%).

Compound	58 nM	116 nM	162.4 nM	185.6 nM	208.8 nM	232 nM	464 nM
6a *	−	−	−	−	−	−	−
**6b**	−	−	−	−	−	−	13.51
**7a**	−	−	−	−	−	5.75	14.99
**7b**	−	−	−	−	−	9.25	17.80
**EDTA-Na_2_**	11.71	20.00	36.37	61.98	86.53	+	+

* Data were previously reported [10], − depicts < 5%, + depicts > 95%.

**Table 11:** Findings from the assessment of cupric ions chelation capacity for the newly catechol derived compounds (%).

Compound	66.66 µM	99.99 µM	133.32 µM	166.65 µM	199.98 µM
6a *	15.04	18.99	21.56	26.05	31.19
**6b**	18.52	25.11	29.73	33.33	38.03
**7a**	16.69	23.15	29.01	34.25	37.12
**7b**	17.18	25.14	30.67	35.14	39.55
**EDTA-Na_2_**	18.54	23.89	29.21	38.01	44.48

* Data were previously reported [10]

**Table 12:** A comparative display of the changes in peak currents and oxidation/reduction potentials for the examined compounds.

Compound	Concentration (μM)	E_ox_ (V)	I_ox_ (μA)	E_ox_ (V)	I_ox_ (μA)	E_Red_ (V)	I_Red_ (μA)
**6a**	250	0.300	23.85	0.54	5.94	0.223	−9.45
	500	0.330	28.84	0.57	8.28	0.255	−14.72
**6b**	250	0.330	19.03	0.533	4.17	0.165	−4.24
	500	0.360	24.96	0.57	7.58	0.200	−6.71
**7a**	250	0.201	7.50	0.418	20.04	0.157	−9.11
	500	0.241	11.16	0.364	39.95	0.223	−16.12
**7b**	250	0.235	16.92	0.487	24.50	0.020	−9.45
	500	0.274	22.75	0.487	33.72	0.244	−17.73

**Table 13:** A comparative illustration of the scavenging impact of the tested substances on ferricyanide.

Sample	Electrochemical Oxidation					Electrochemical Reduction				
	E_ox_ (V)	I_ox_ (μA)	E_ox_ (V)	I_ox_ (μA)	E_ox_ (V)	I_ox_ (μA)	E_red_ (V)	I_red_ (μA)	E_red_ (V)	I_red_ (μA)
FC * 500 μM	0.235	4.43	-	-	-	-	0.140	−4.69	-	-
**6a** 250 μM	-	-	0.301	23.85	0.540	5.95	-	-	0.223	−9.45
**6a** 250 μM + FC * 500 μM	0.182	2.46	0.348	19.94	0.589	5.72	0.159	−15.55	0.250	−2.22
**6b** 250 μM	-	-	0.331	19.03	0.533	4.17	-	-	0.165	−4.24
**6b** 250 μM + FC * 500 μM	0.172	2.82	0.359	20.64	0.570	3.91	0.130	−11.12	-	-
**7a** 250 μM	-	-	0.201	7.51	0.437	10.13	-	-	0.157	−9.11
**7a** 250 μM + FC * 500 μM	0.147	1.07	0.255	1.55	0.653	13.58	−0.035	−21.03	-	-
**7b** 250 μM	-	-	0.235	16.92	0.499	15.61	-	-	0.020	−9.45
**7b** 250 μM + FC * 500 μM	0.130	0.39	0.252	5.34	0.467	12.72	0.093	−12.58	-	-

* FC = Ferricyanide.

**Table 14:** A comparative display of the scavenging impact of the tested compounds on H_2_O_2_.

Sample	E_ox_ (V)	I_ox_ (μA)	∆I_ox_ (μA)
H_2_O_2_ 500 μM	0.052	339.50	-
H_2_O_2_ 500 μM + **6a** 250 μM	0.091	45.21	294.29
H_2_O_2_ 500 μM + **6b** 250 μM	0.147	43.67	295.83
H_2_O_2_ 500 μM + **7a** 250 μM	0.071	176.21	163.29
H_2_O_2_ 500 μM + **7b** 250 μM	0.093	105.24	234.26

**Table 15:** A comparative display of the scavenging impact of the 4 tested substances on TEMPO radical.

Sample	Oxidation Signal of TEMPO		Reduction Signal of TEMPO	
	E_ox_ (V)	I_ox_ (μA)	E_red_ (V)	I_red_ (μA)
TEMPO 2 mM	0.301	13.81	0.232	−22.09
TEMPO 2 mM + ascorbic acid 250 μM	0.267	1.44	0.301	−20.01
TEMPO 2 mM + tyrosine 250 μM (60 min)	0.289	13.28	0.223	−23.21
TEMPO 2 mM + **6a** 250 μM (60 min)	0.299	1.59	0.334	−20.66
TEMPO 2 mM + **6b** 250 μM (60 min)	0.108	0.10	0.333	−11.45
TEMPO 2 mM + **7a** 250 μM (60 min)	0.120	0.24	0.325	−22.12
TEMPO 2 mM + **7b** 250 μM (60 min)	0.242	4.50	0.343	−19.75

**Table 16:** IC_50_ values of the tested compounds against human normal and cancer cell lines (µg/mL).

Cell Line	6a	6b	7a	7b	Ascorbic Acid
HaCaT	158.29	26.1	219.26	23.75	1091.63
BJ	26.98	42.37	104.42	72.86	68.22
A375	229.27	23.11	263.46	82.92	538.48
A549	5.45	206.42	88.62	42.14	1597.05

## Data Availability

Not applicable.

## Supplementary Materials

The following supporting information can be downloaded at: https://www.mdpi.com/article/10.3390/ijms241713277/s1.

## References

[1] Pizzino G., Irrera N., Cucinotta M., Pallio G., Mannino F., Arcoraci V., Squadrito F., Altavilla D., Bitto A. (2017). Oxidative Stress: Harms and Benefits for Human Health. Oxid. Med. Cell. Longev..

[2] Sharifi-Rad M., Anil Kumar N.V., Zucca P., Varoni E.M., Dini L., Panzarini E., Rajkovic J., Tsouh Fokou P.V., Azzini E., Peluso I. (2020). Lifestyle, Oxidative Stress, and Antioxidants: Back and Forth in the Pathophysiology of Chronic Diseases. Front. Physiol..

[3] Forman H.J., Zhang H. (2021). Targeting oxidative stress in disease: Promise and limitations of antioxidant therapy. Nat. Rev. Drug Discov..

[4] García-Sánchez A., Miranda-Díaz A.G., Cardona-Muñoz E.G. (2020). The Role of Oxidative Stress in Physiopathology and Pharmacological Treatment with Pro- and Antioxidant Properties in Chronic Diseases. Oxid. Med. Cell. Longev..

[5] Sies H., Berndt C., Jones D.P. (2017). Oxidative Stress. Annu. Rev. Biochem..

[6] Cory H., Passarelli S., Szeto J., Tamez M., Mattei J. (2018). The Role of Polyphenols in Human Health and Food Systems: A Mini-Review. Front. Nutr..

[7] Rana A., Samtiya M., Dhewa T., Mishra V., Aluko R.E. (2022). Health benefits of polyphenols: A concise review. J. Food Biochem..

[8] Cutrim C.S., Cortez M.A.S. (2018). A review on polyphenols: Classification, beneficial effects and their application in dairy products. Int. J. Dairy Technol..

[9] Hussain T., Tan B., Yin Y., Blachier F., Tossou M.C.B., Rahu N. (2016). Oxidative Stress and Inflammation: What Polyphenols Can Do for Us?. Oxid. Med. Cell. Longev..

[10] Mic M., Pîrnău A., Floare C.G., Borlan R., Focsan M., Oniga O., Bogdan M., Vlase L., Oniga I., Marc G. (2022). Antioxidant Activity Evaluation and Assessment of the Binding Affinity to HSA of a New Catechol Hydrazinyl-Thiazole Derivative. Antioxidants.

[11] Tayade K., Yeom G.-S., Sahoo S.K., Puschmann H., Nimse S.B., Kuwar A. (2022). Exploration of Molecular Structure, DFT Calculations, and Antioxidant Activity of a Hydrazone Derivative. Antioxidants.

[12] Grozav A., Porumb I.-D., Găină L., Filip L., Hanganu D. (2017). Cytotoxicity and Antioxidant Potential of Novel 2-(2-((1H-indol-5yl)methylene)-hydrazinyl)-thiazole Derivatives. Molecules.

[13] Swesi A.T., Farina Y., Kassim M., Ng S.W. (2006). 2,3-Dihydroxybenzaldehyde thiosemicarbazone hemihydrate. Acta Crystallogr. Sect. E Struct. Reports Online.

[14] Palanimuthu D., Poon R., Sahni S., Anjum R., Hibbs D., Lin H.-Y., Bernhardt P.V., Kalinowski D.S., Richardson D.R. (2017). A novel class of thiosemicarbazones show multi-functional activity for the treatment of Alzheimer’s disease. Eur. J. Med. Chem..

[15] Gatti A., Habtemariam A., Romero-Canelón I., Song J.-I., Heer B., Clarkson G.J., Rogolino D., Sadler P.J., Carcelli M. (2018). Half-Sandwich Arene Ruthenium(II) and Osmium(II) Thiosemicarbazone Complexes: Solution Behavior and Antiproliferative Activity. Organometallics.

[16] Marc G., Stana A., Oniga S.D., Pîrnău A., Vlase L., Oniga O. (2019). New Phenolic Derivatives of Thiazolidine-2,4-dione with Antioxidant and Antiradical Properties: Synthesis, Characterization, In Vitro Evaluation, and Quantum Studies. Molecules.

[17] Marc G., Stana A., Franchini A.H., Vodnar D.C., Barta G., Tertiş M., Şanta I., Cristea C., Pîrnău A., Ciorîţă A. (2021). Phenolic Thiazoles with Antioxidant and Antiradical Activity. Synthesis, In Vitro Evaluation, Toxicity, Electrochemical Behavior, Quantum Studies and Antimicrobial Screening. Antioxidants.

[18] Pele R., Marc G., Stana A., Ionuț I., Nastasă C., Tiperciuc B., Oniga I., Pîrnău A., Vlase L., Oniga O. (2022). Synthesis of New Phenolic Derivatives of Quinazolin-4(3H)-One as Potential Antioxidant Agents—In Vitro Evaluation and Quantum Studies. Molecules.

[19] Pele R., Marc G., Ionuț I., Nastasă C., Fizeșan I., Pîrnău A., Vlase L., Palage M., Oniga S., Oniga O. (2022). Antioxidant and Cytotoxic Activity of New Polyphenolic Derivatives of Quinazolin-4(3H)-one: Synthesis and In Vitro Activities Evaluation. Pharmaceutics.

[20] Guan S., Wang L., Xu S.-M., Yang D., Waterhouse G.I.N., Qu X., Zhou S. (2019). Vacancy-enhanced generation of singlet oxygen for photodynamic therapy. Chem. Sci..

[21] Carp O.E., Moraru A., Pinteala M., Arvinte A. (2021). Electrochemical behaviour of piperine. Comparison with control antioxidants. Food Chem..

[22] Gorjanović S.Z., Novaković M.M., Potkonjak N.I., LeskoŠek-Čukalović I., Sužnjević D.Z. (2010). Application of a Novel Antioxidative Assay in Beer Analysis and Brewing Process Monitoring. J. Agric. Food Chem..

[23] Sužnjević D.Ž., Pastor F.T., Gorjanović S.Ž. (2011). Polarographic study of hydrogen peroxide anodic current and its application to antioxidant activity determination. Talanta.

[24] Pandithavidana D.R., Jayawardana S.B. (2019). Comparative Study of Antioxidant Potential of Selected Dietary Vitamins; Computational Insights. Molecules.

[25] Antonijević M.R., Simijonović D.M., Avdović E.H., Ćirić A., Petrović Z.D., Marković J.D., Stepanić V., Marković Z.S. (2021). Green One-Pot Synthesis of Coumarin-Hydroxybenzohydrazide Hybrids and Their Antioxidant Potency. Antioxidants.

[26] Liu Y.-S., Zhang G.-Y., Hou Y. (2023). Theoretical and Experimental Investigation of the Antioxidation Mechanism of Loureirin C by Radical Scavenging for Treatment of Stroke. Molecules.

[27] Nantasenamat C., Isarankura-Na-Ayudhya C., Naenna T., Prachayasittikul V. (2008). Prediction of bond dissociation enthalpy of antioxidant phenols by support vector machine. J. Mol. Graph. Model..

[28] Zhu Q., Zhang X.-M., Fry A.J. (1997). Bond dissociation energies of antioxidants. Polym. Degrad. Stab..

[29] Chen Y., Xiao H., Zheng J., Liang G. (2015). Structure-Thermodynamics-Antioxidant Activity Relationships of Selected Natural Phenolic Acids and Derivatives: An Experimental and Theoretical Evaluation. PLoS ONE.

[30] Vo Q.V., Nam P.C., Thong N.M., Trung N.T., Phan C.-T.D., Mechler A. (2019). Antioxidant Motifs in Flavonoids: O–H versus C–H Bond Dissociation. ACS Omega.

[31] Amić A., Mastiľák Cagardová D. (2022). DFT Study of the Direct Radical Scavenging Potency of Two Natural Catecholic Compounds. Int. J. Mol. Sci..

[32] Linciano P., Moraes C.B., Alcantara L.M., Franco C.H., Pascoalino B., Freitas-Junior L.H., Macedo S., Santarem N., Cordeiro-da-Silva A., Gul S. (2018). Aryl thiosemicarbazones for the treatment of trypanosomatidic infections. Eur. J. Med. Chem..

[33] Bernstein J., Yale H.L., Losee K., Holsing M., Martins J., Lott W.A. (1951). The Chemotherapy of Experimental Tuberculosis. III. The Synthesis of Thiosemicarbazones and Related Compounds 1,2. J. Am. Chem. Soc..

[34] Benzie I.F.F., Strain J.J., Packer L. (1999). Ferric reducing/antioxidant power assay: Direct measure of total antioxidant activity of biological fluids and modified version for simultaneous measurement of total antioxidant power and ascorbic acid concentration. Methods in Enzymology.

[35] Ganea I.-V., Nan A., Ciorîță A., Turcu R., Baciu C. (2022). Responsiveness assessment of cell cultures exposed to poly(tartaric acid) and its corresponding magnetic nanostructures. J. Mol. Struct..

[36] De Jong N.W., Ploscariu N.T., Ramyar K.X., Garcia B.L., Herrera A.I., Prakash O., Katz B.B., Leidal K.G., Nauseef W.M., van Kessel K.P. (2018). A structurally dynamic N-terminal region drives function of the staphylococcal peroxidase inhibitor (SPIN). J. Biol. Chem..

[37] Shangari N., O’Brien P.J. (2006). Catalase Activity Assays. Curr. Protoc. Toxicol..

