# Supplementary material for: Discovery of New Hydrazone-Thiazole Polyphenolic Antioxidants through Computer-Aided Design and In Vitro Experimental Validation
Source: Int J Mol Sci. 2023 Aug 26;24(17):13277. doi: 10.3390/ijms241713277 (PMC10487687; doi:10.3390/ijms241713277)

International Journal of *Molecular Sciences*

MDPI

Supplementary Material

Discovery of New Hydrazone-Thiazole Polyphenolic Antioxidants through Computer-Aided Design and In Vitro Experimental Validation

Gabriel Marc [1], Anca Stana [1,*], Mihaela Tertiş [2], Cecilia Cristea [2], Alexandra Ciorîţă [3], Ștefan-Mihai Drăgan [4], Vlad-Alexandru Toma [4,5], Raluca Borlan [6], Monica Focșan [6], Adrian Pîrnău [3], Laurian Vlase [7], Smaranda Oniga [8] and Ovidiu Oniga [1]

1 Department of Pharmaceutical Chemistry, "Iuliu Hațieganu" University of Medicine and Pharmacy, 41 Victor Babeș Street, RO-400012 Cluj-Napoca, Romania; marc.gabriel@umfcluj.ro (G.M.); stana.anca@umfcluj.ro (A.S.); ooniga@umfcluj.ro (O.O.)
2 Department of Analytical Chemistry, Faculty of Pharmacy, "Iuliu Haţieganu" University of Medicine and Pharmacy, 4 Louis Pasteur Street, RO-400349 Cluj-Napoca, Romania; mihaela.tertis@umfcluj.ro (M.T.); ccristea@umfcluj.ro (C.C.)
3 National Institute for Research and Development of Isotopic and Molecular Technologies, 67-103 Donath Street, RO-400293 Cluj-Napoca, Romania; alexandra.ciorita@itim-cj.ro (A.C.); apirnau@itim-cj.ro (A.P.)
4 Department of Molecular Biology and Biotechnology, Faculty of Biology and Geology, Babeș-Bolyai University, Clinicilor Street No. 4-7, RO-400006 Cluj-Napoca, Romania; stefandragan81@gmail.com (Ș.-M.D.); vlad.toma@ubbcluj.ro (V.-A.T.)
5 Institute of Biological Research, Republicii Street No. 48, Branch of NIRDBS Bucharest, RO-400015 Cluj-Napoca, Romania
6 Nanobiophotonics and Laser Microspectroscopy Centre, Interdisciplinary Research Institute in Bio-Nano-Sciences, Babeș-Bolyai University, RO-400084 Cluj-Napoca, Romania; raluca.borlan@ubbcluj.ro (R.B.); monica.iosin@ubbcluj.ro (M.F.)
7 Department of Pharmaceutical Technology and Biopharmaceutics, "Iuliu Hațieganu" University of Medicine and Pharmacy, 41 Victor Babeș Street, RO-400012 Cluj-Napoca, Romania; laurian.vlase@umfcluj.ro (L.V.)
8 Department of Therapeutic Chemistry, "Iuliu Hațieganu" University of Medicine and Pharmacy, 12 Ion Creangă Street, RO-400010 Cluj-Napoca, Romania; smaranda.oniga@umfcluj.ro (S.O.)
* Correspondence: stana.anca@umfcluj.ro or teodora_anca@yahoo.com; Tel.: +40-264-450-529

Citation: Marc, G.; Stana, A.; Tertiş, M.; Cristea, C.; Ciorîţă, A.; Drăgan, Ș.-M.; Toma, V.-A.; Borlan, R.; Focşan, M.; Pîrnău, A.; et al. Discovery of New Hydrazone-Thiazole Polyphenolic Antioxidants through Computer-Aided Design and In Vitro Experimental Validation. *Int. J. Mol. Sci.* **2023**, *24*, x. https://doi.org/10.3390/xxxxx

Academic Editor(s): Name

Received: 28 July 2023
Revised: 12 August 2023
Accepted: date
Published: date

Copyright: © 2023 by the authors. Submitted for possible open access publication under the terms and conditions of the Creative Commons Attribution (CC BY) license (https://creativecommons.org/licenses/by/4.0/).

1. Figures

1.1. The IR spectra of the compounds

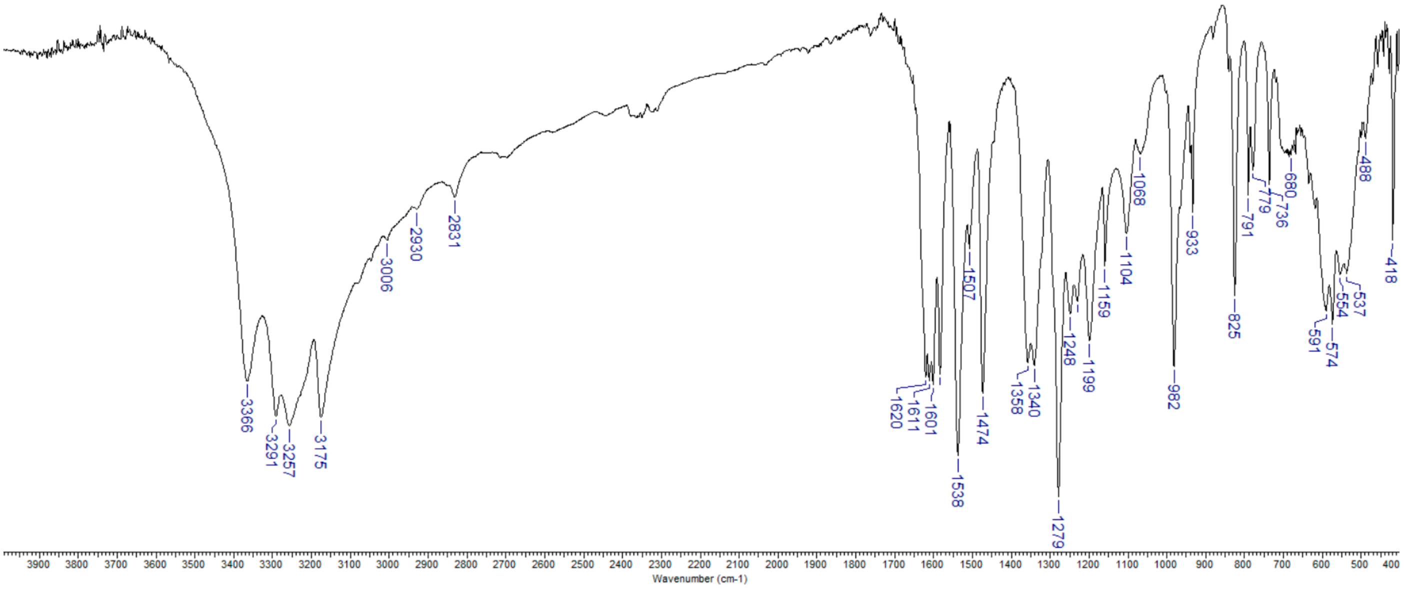

Figure S1. The IR spectrum for the compound **3b.**

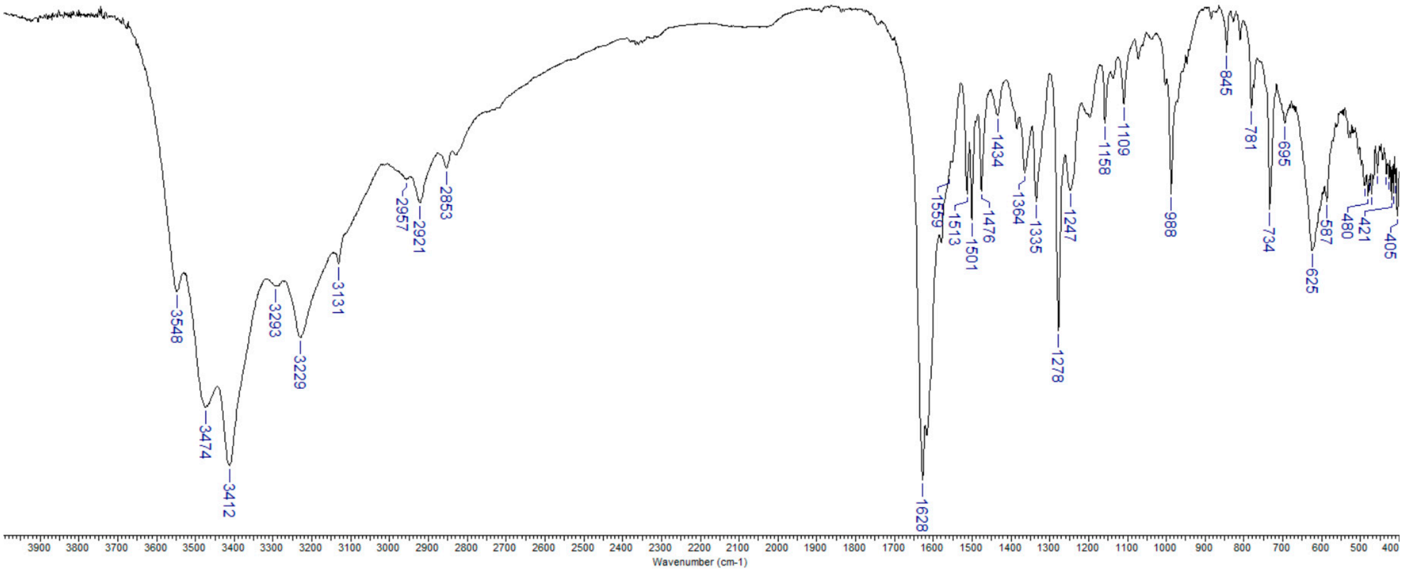

Figure S2. The IR spectrum of the compound **6b.**

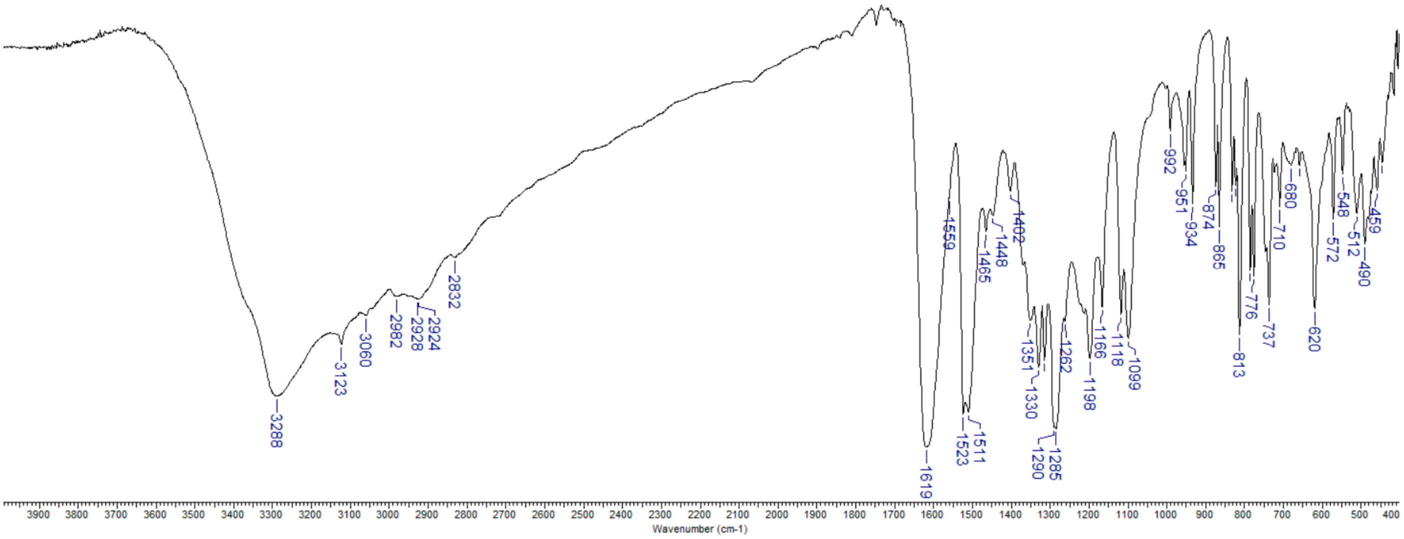

Figure S3. The IR spectrum of the compound **7a.**

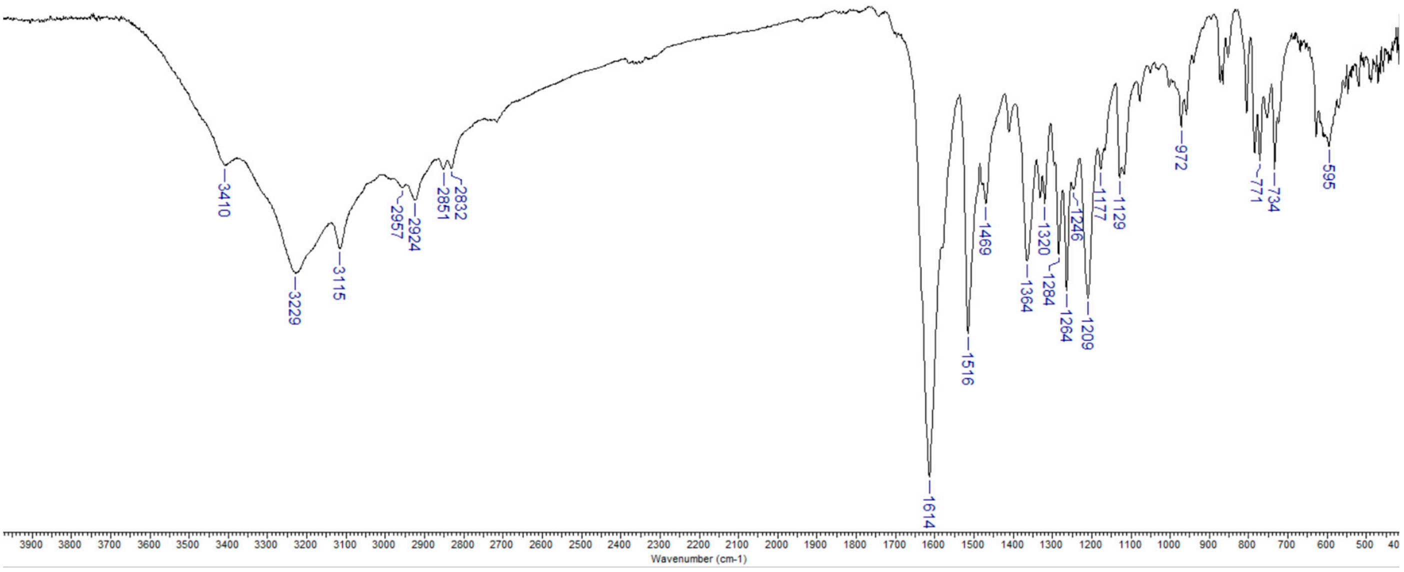

Figure S4. The IR spectrum of the compound **7b.**

1.2. The mass spectra or the compounds

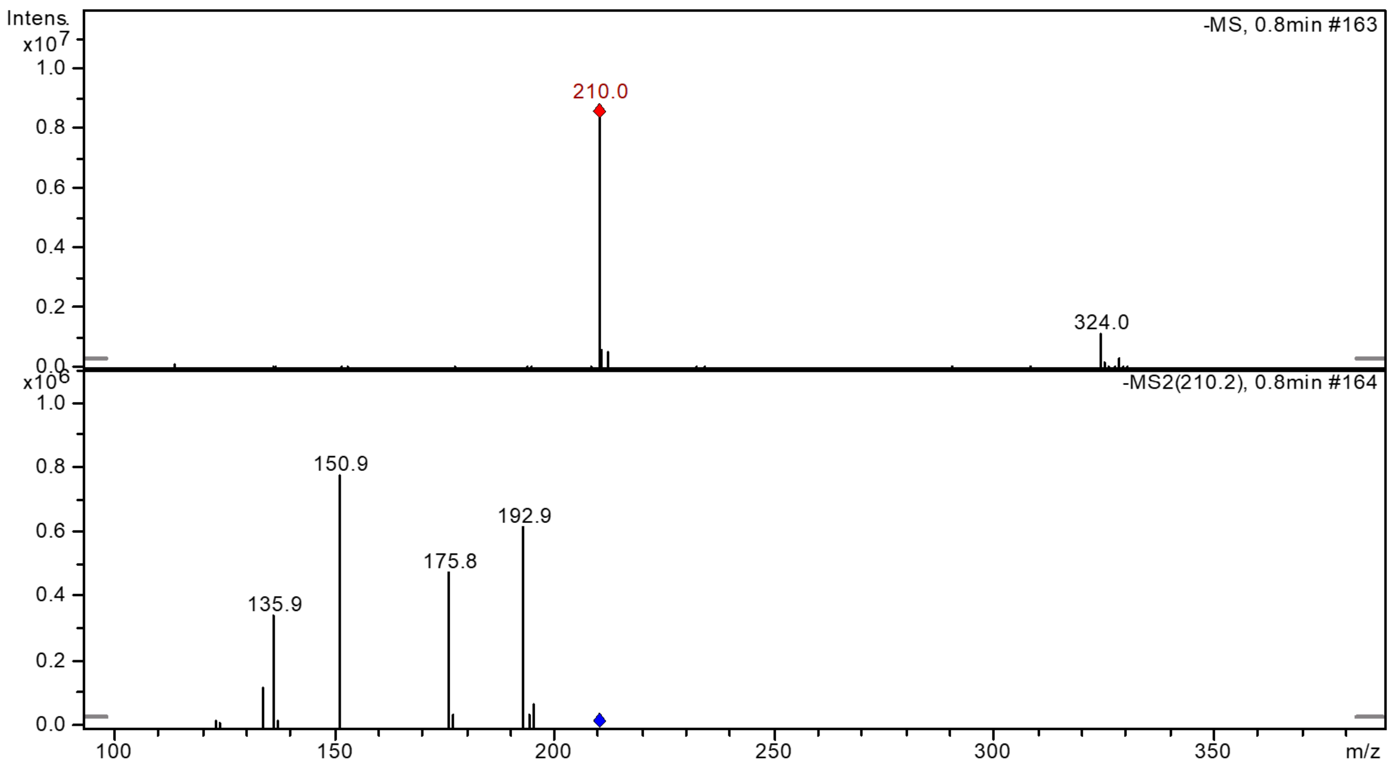

Figure S5. The mass spectrum of the compound **3b.**

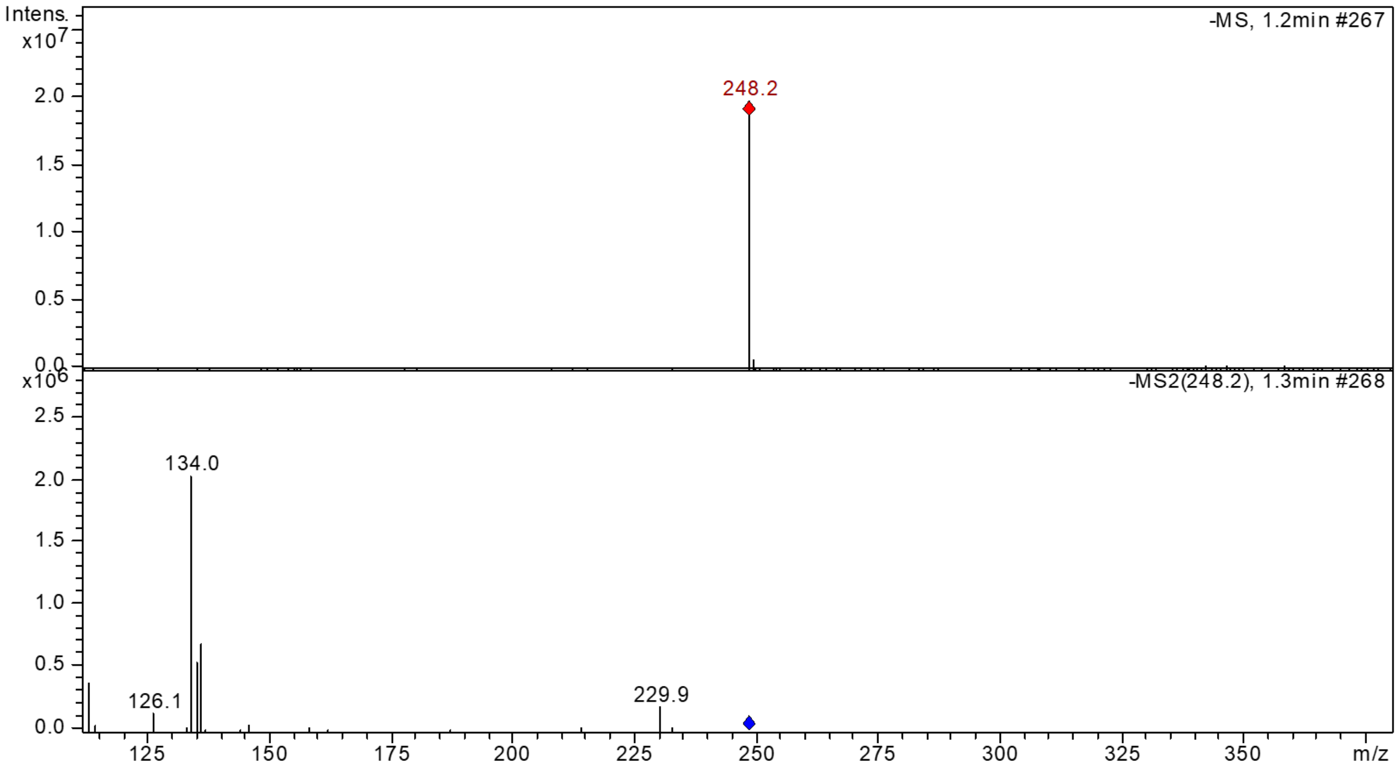

Figure S6. The mass spectrum of the compound **6b.**

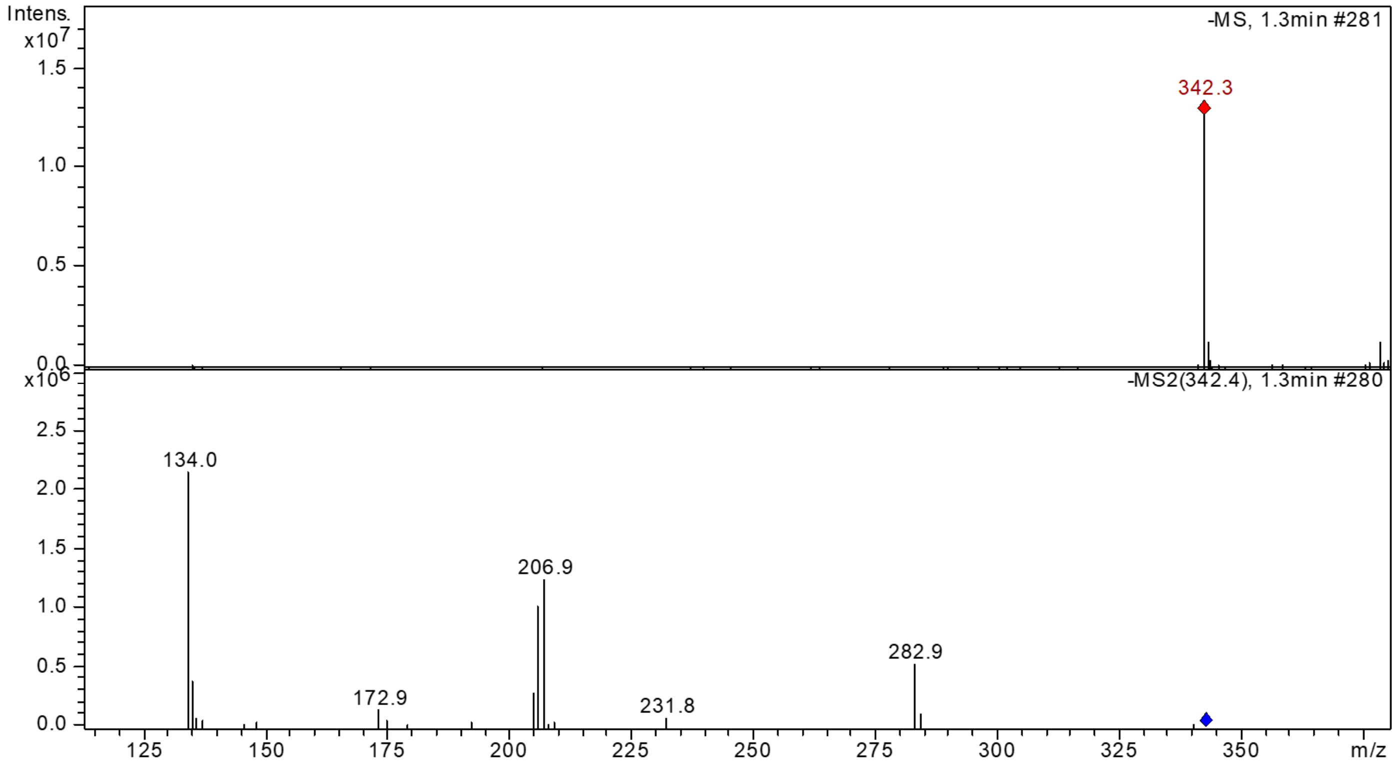

Figure S7. The mass spectrum of the compound **7a.**

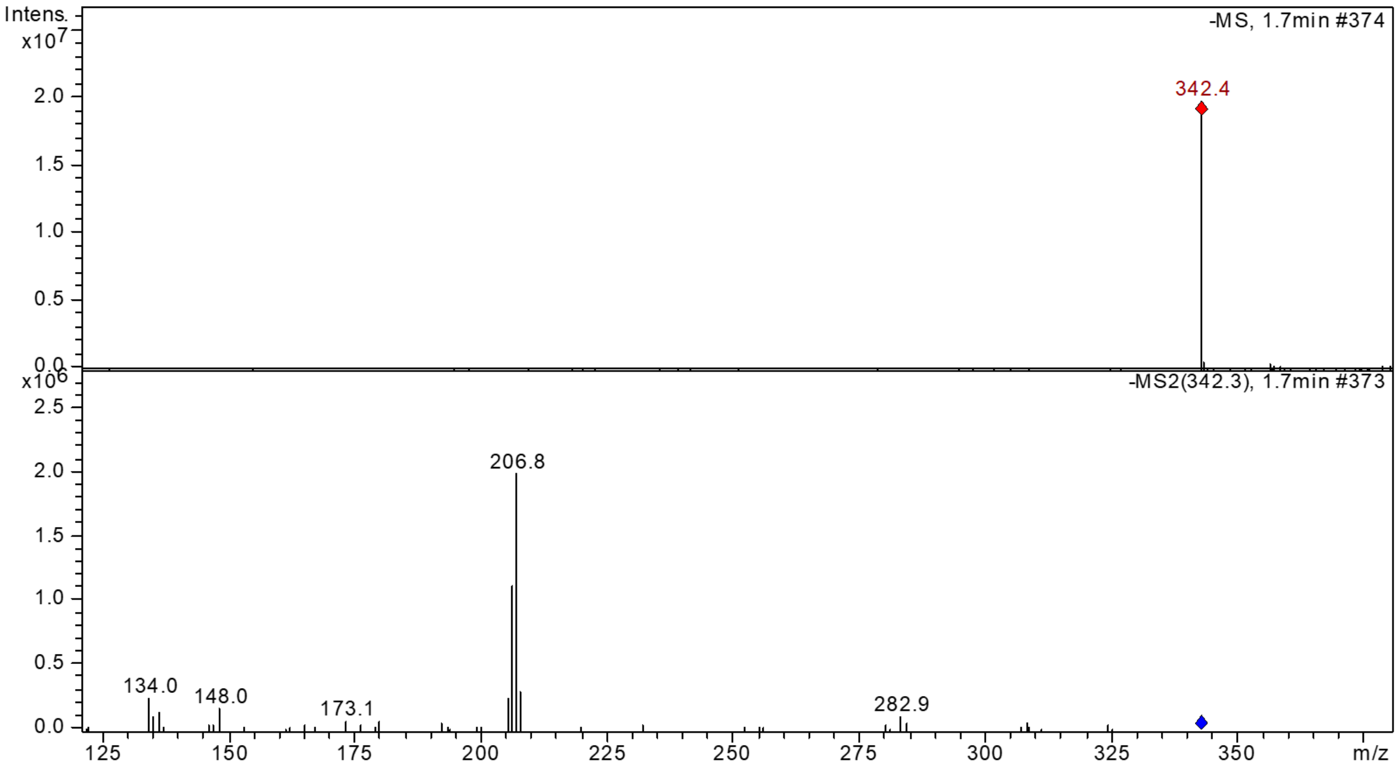

Figure S8. The mass spectrum of the compound **7b.**

1.3. The ¹H NMR spectra of the compounds

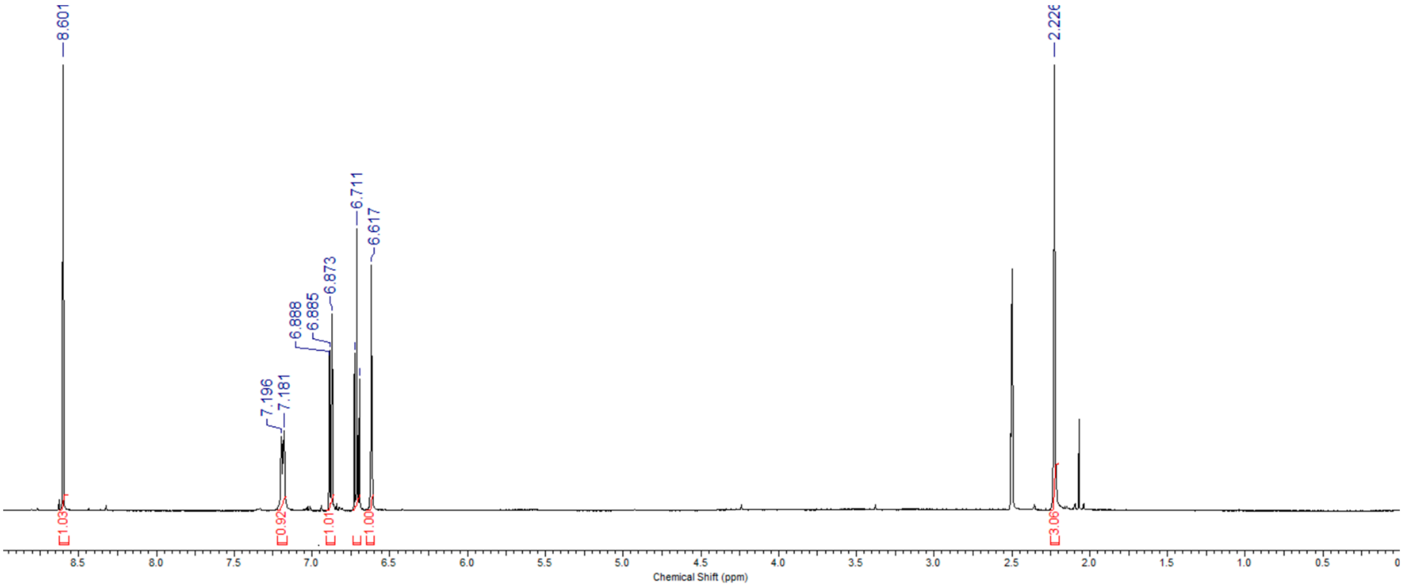

Figure S9. The ¹H NMR spectrum of the compound **6b.**

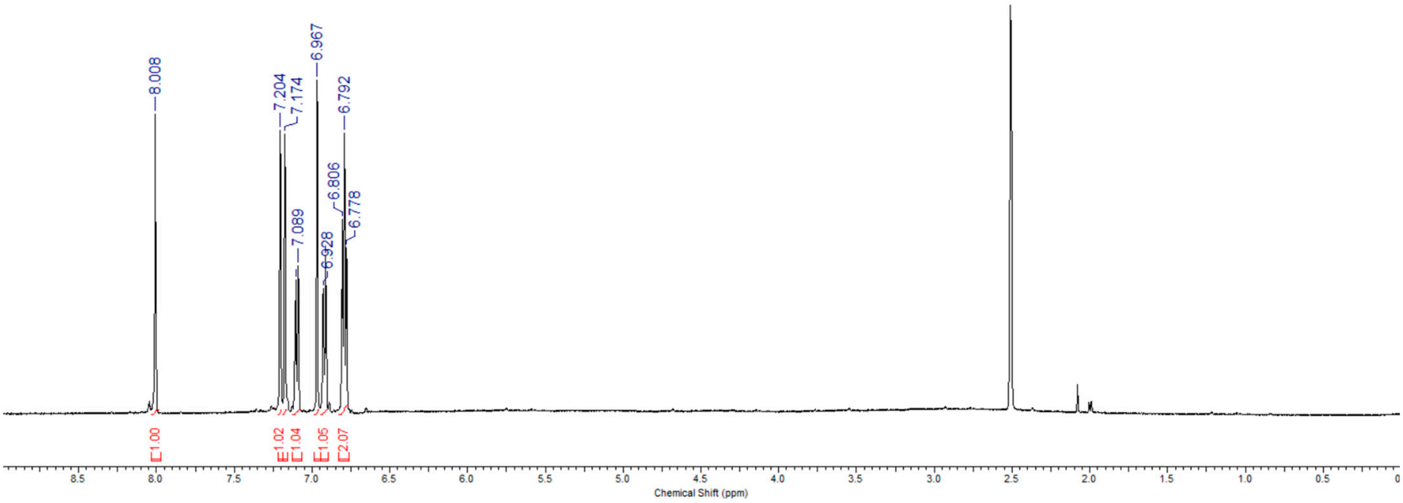

Figure S10. The ¹H NMR spectrum of the compound **7a.**

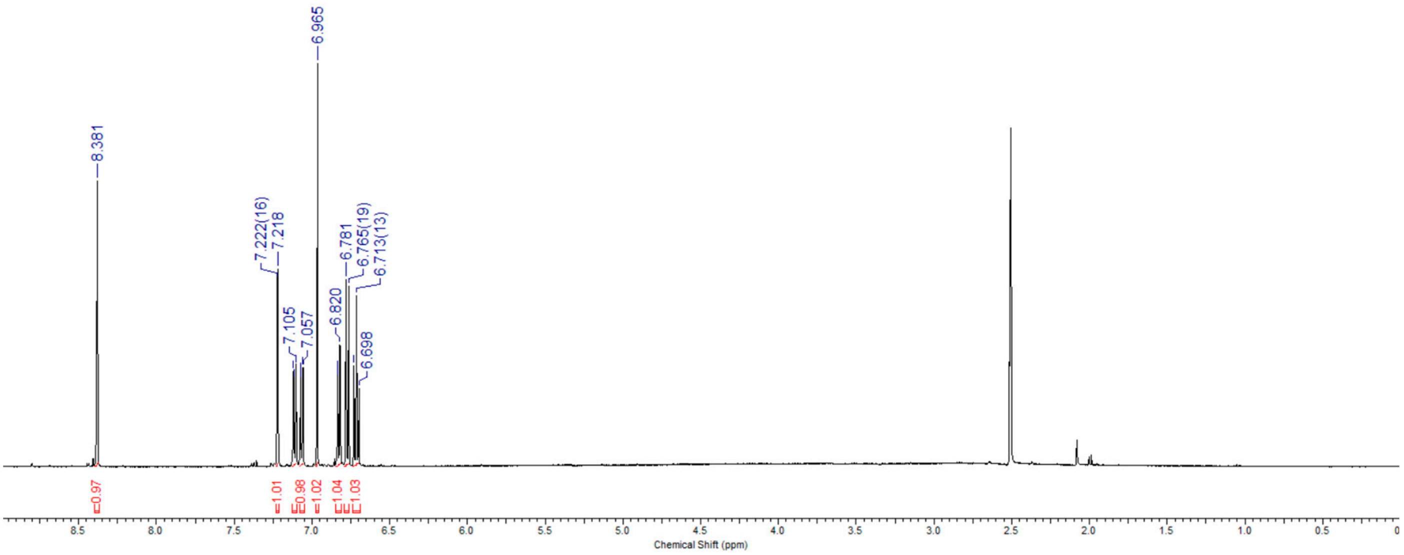

Figure S11. The ^{1}H NMR spectrum of the compound **7b.**

1.4. The ^{13}C NMR spectra of the compounds

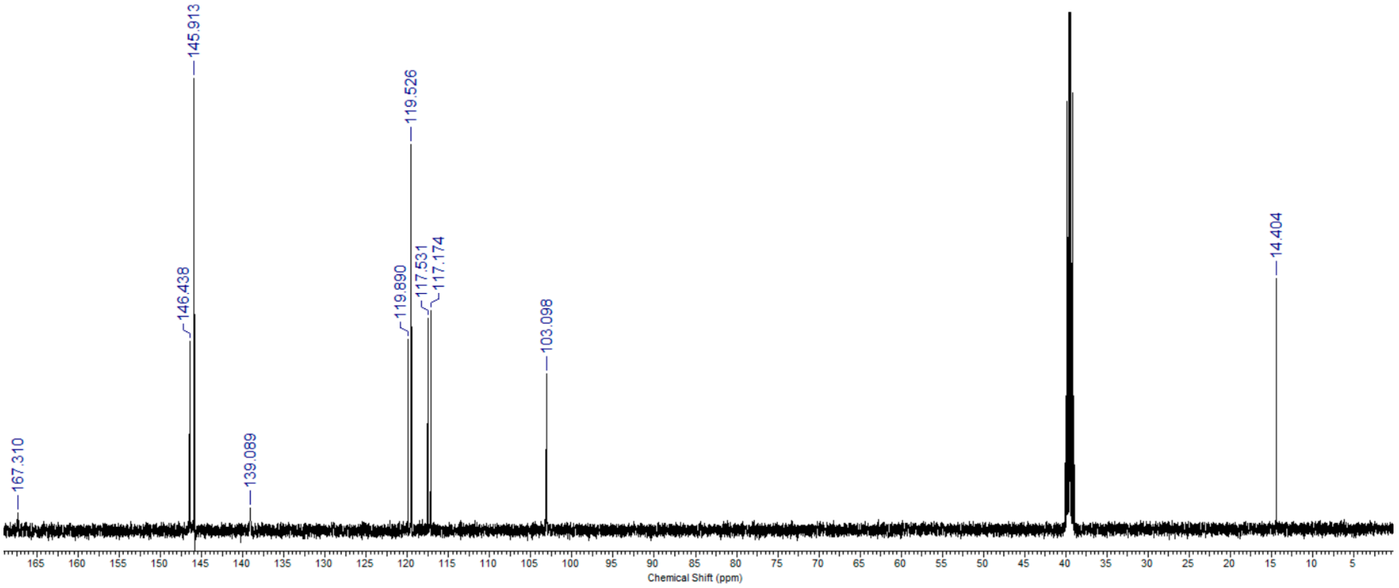

Figure S12. The ^{13}C NMR spectrum of the compound **6b.**

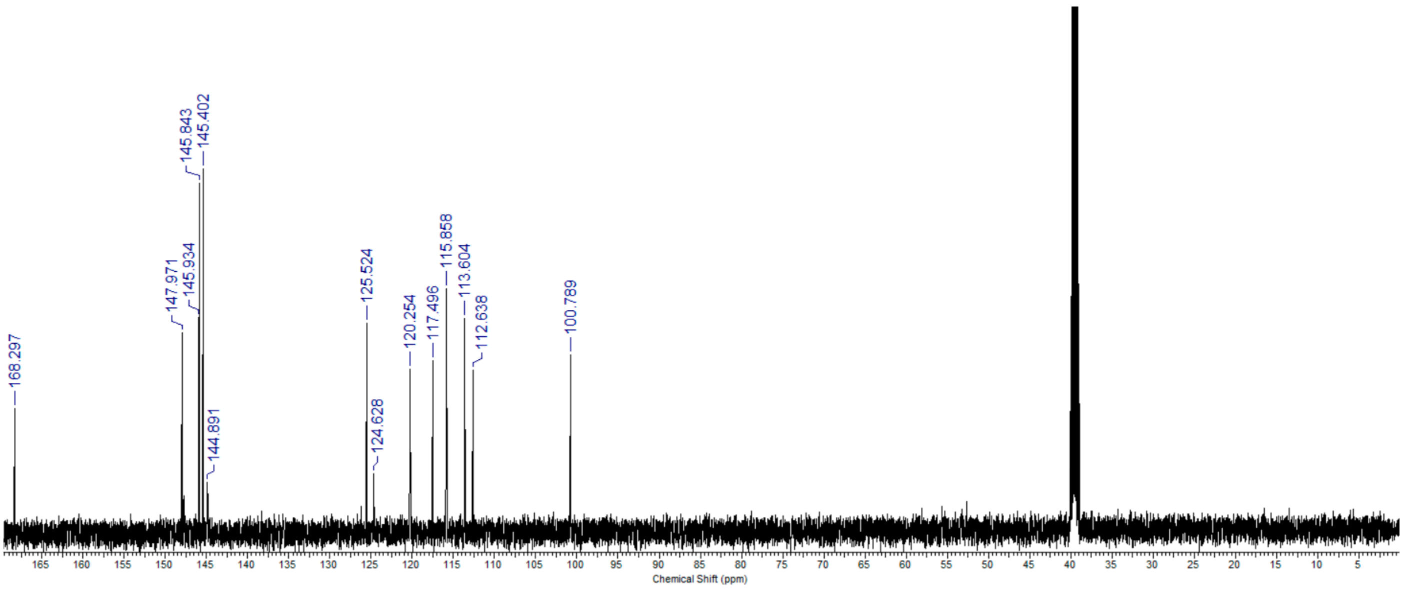

Figure S13. The ^{13}C NMR spectrum of the compound **7a.**

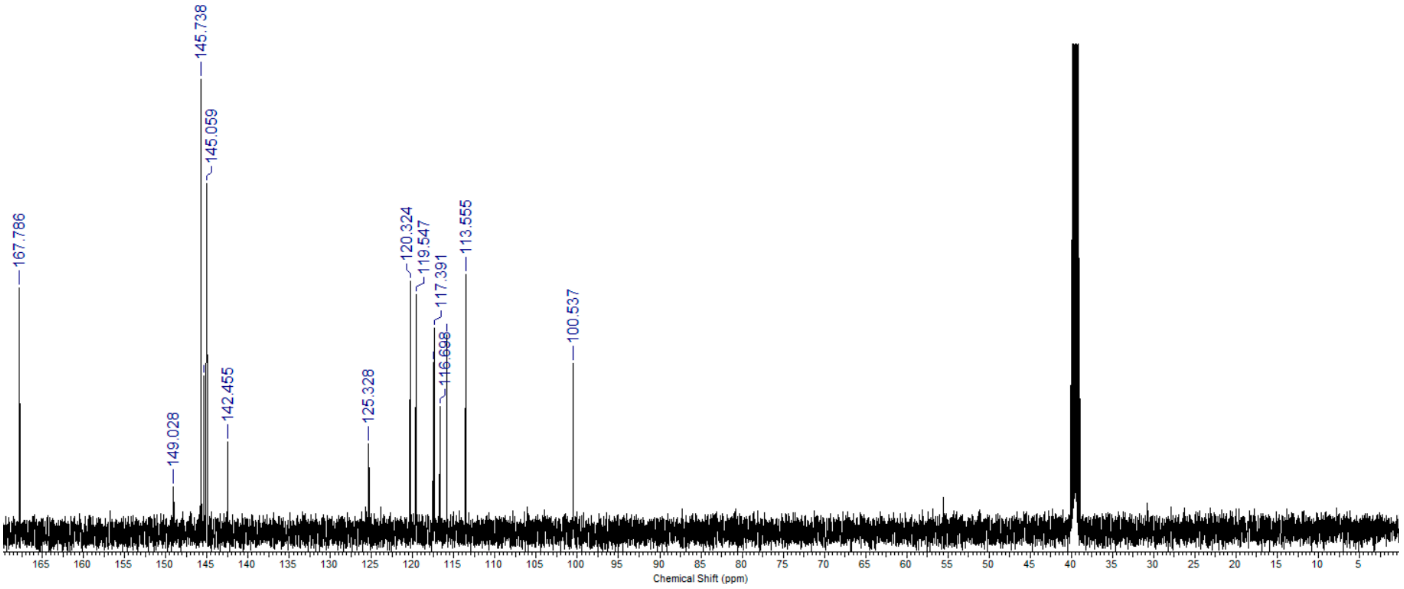

Figure S14. The ^{13}C NMR spectrum of the compound **7b.**

2. Tables

Table S1. HOMO and LUMO frontier orbitals of compounds **6a-b** and **7a-b**.

Compound	HOMO	LUMO
**6a**	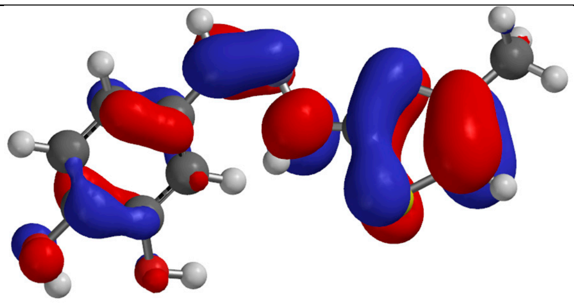<br>**E_{HOMO} = -5.30 eV**	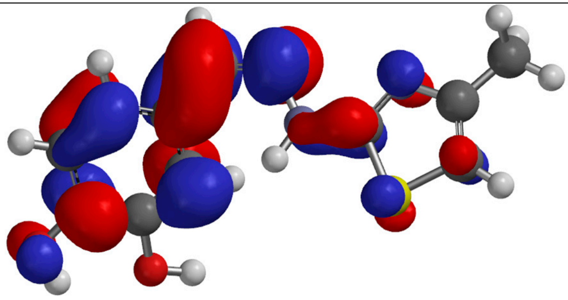<br>**E_{LUMO} = -1.13 eV**
**6b**	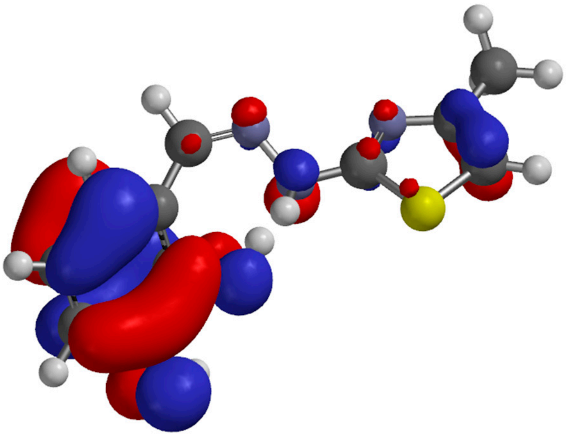<br>**E_{HOMO} = -5.87 eV**	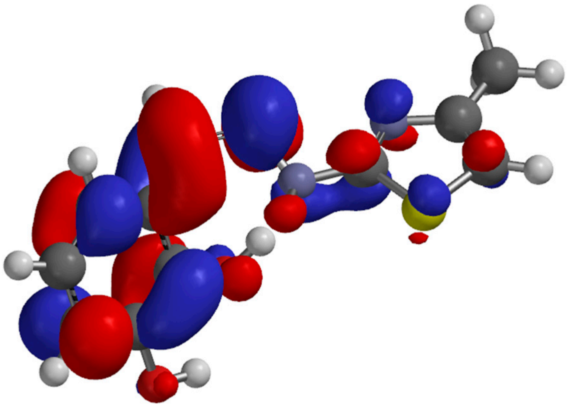<br>**E_{LUMO} = -1.33 eV**
**7a**	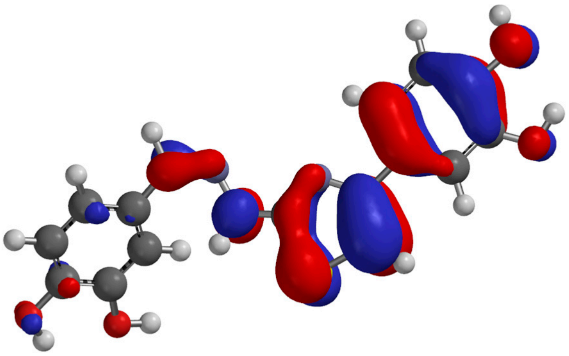<br>**E_{HOMO} = -5.08 eV**	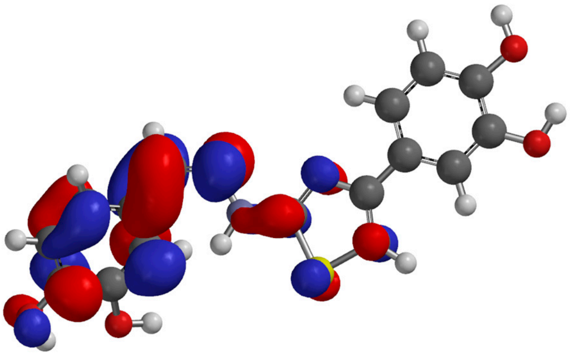<br>**E_{LUMO} = -1.16 eV**
**7b**	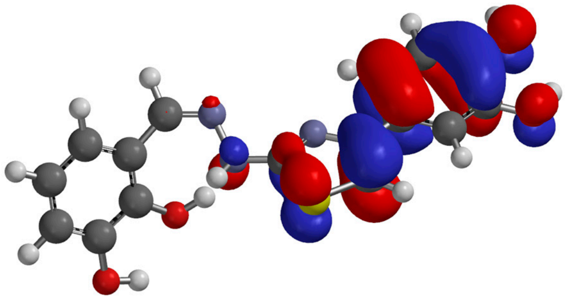<br>**E_{HOMO} = -5.41 eV**	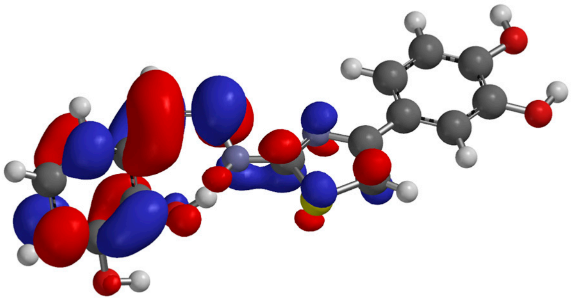<br>**E_{LUMO} = -1.35 eV**

Table S2. Electrostatic potential maps of compounds **6a-b** and **7a-b**.

Compound	Electrostatic potential map
**6a**	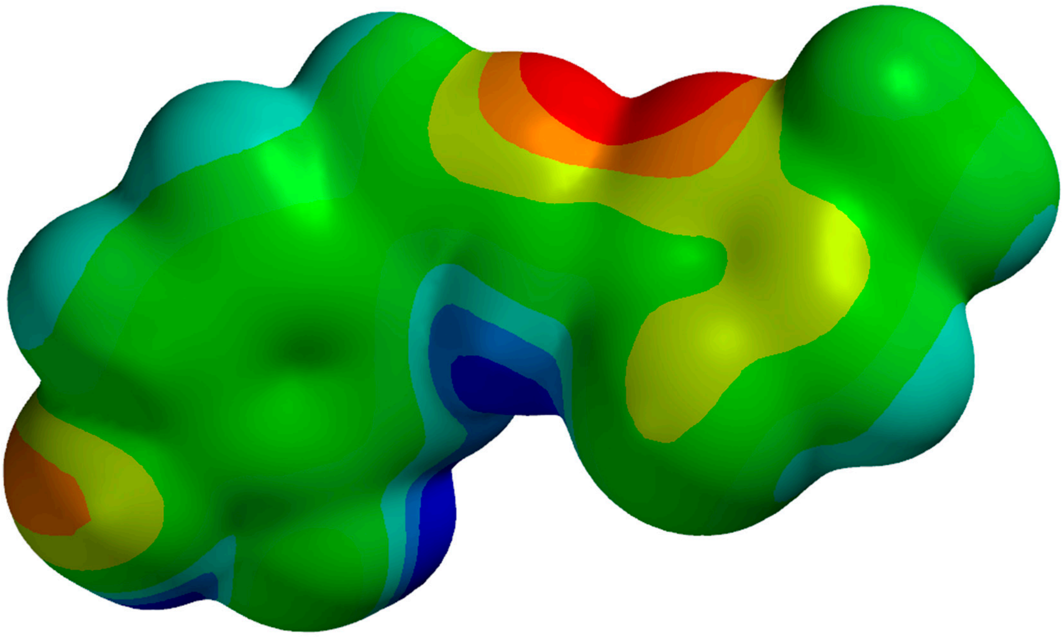
**6b**	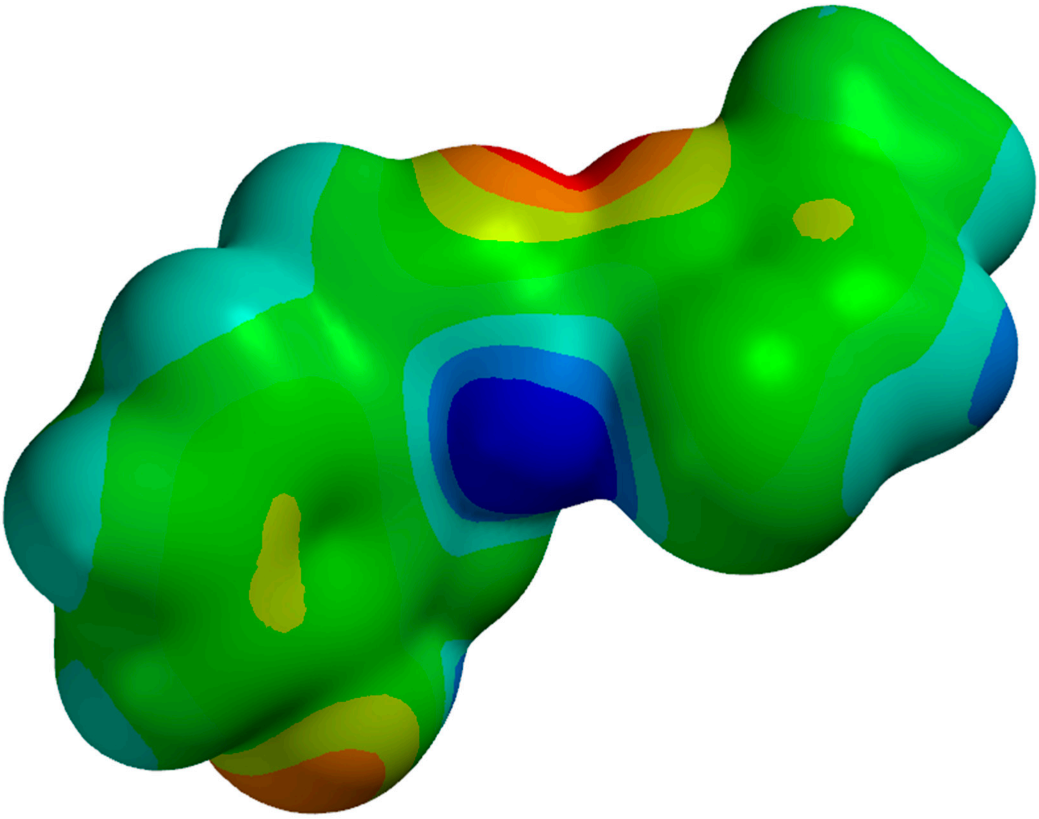

7a

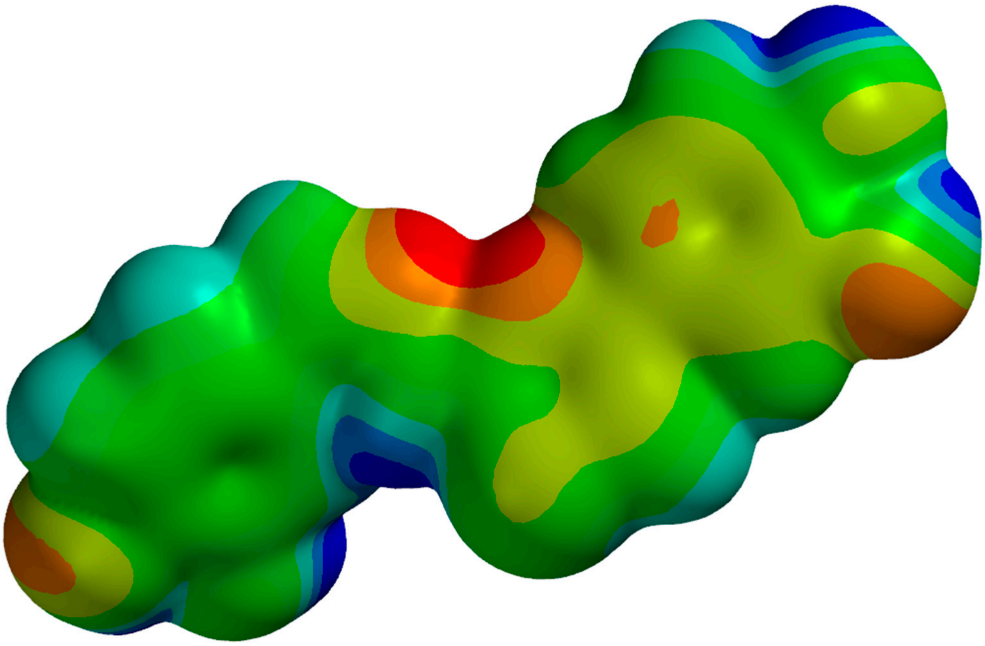

7b

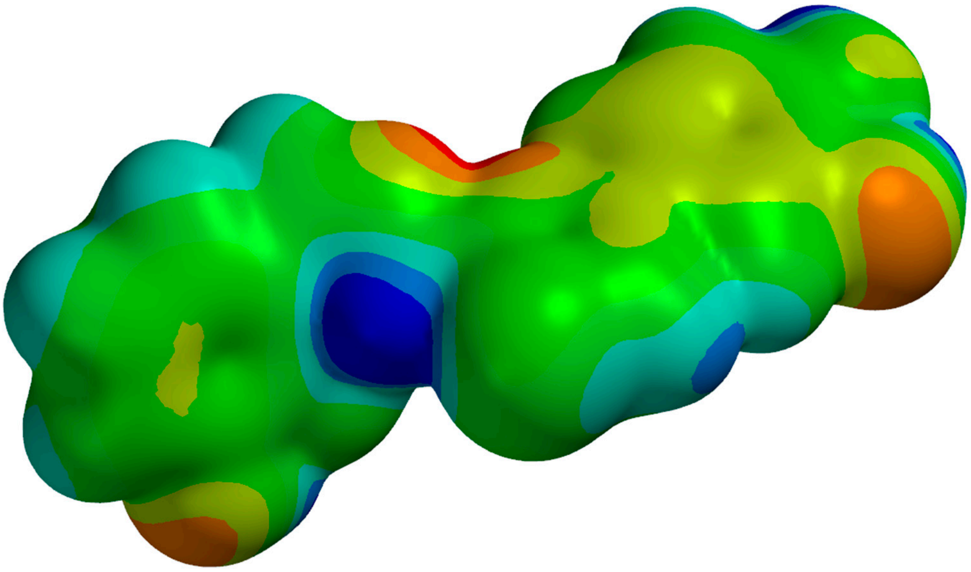

Supplement: Supplementary file 1 [file ijms-24-13277-s001.zip › ijms-2557222-supplementary.pdf]
